# Cortical–hippocampal coupling during manifold exploration in motor cortex

**DOI:** 10.1038/s41586-022-05533-z

**Published:** 2022-12-14

**Authors:** Jaekyung Kim, Abhilasha Joshi, Loren Frank, Karunesh Ganguly

**Affiliations:** 1 Neurology Service, San Francisco Veterans Affairs Medical Center, San Francisco, CA USA; 2 Department of Neurology, University of California, San Francisco, San Francisco, CA USA; 3 HHMI and Departments of Physiology and Psychiatry, University of California, San Francisco, San Francisco, CA USA

**Keywords:** Consolidation, Motor cortex

## Abstract

Systems consolidation—a process for long-term memory stabilization—has been hypothesized to occur in two stages^1–4^. Whereas new memories require the hippocampus^5–9^, they become integrated into cortical networks over time^10–12^, making them independent of the hippocampus. How hippocampal–cortical dialogue precisely evolves during this and how cortical representations change in concert is unknown. Here, we use a skill learning task^13,14^ to monitor the dynamics of cross-area coupling during non-rapid eye movement sleep along with changes in primary motor cortex (M1) representational stability. Our results indicate that precise cross-area coupling between hippocampus, prefrontal cortex and M1 can demarcate two distinct stages of processing. We specifically find that each animal demonstrates a sharp increase in prefrontal cortex and M1 sleep slow oscillation coupling with stabilization of performance. This sharp increase then predicts a drop in hippocampal sharp-wave ripple (SWR)–M1 slow oscillation coupling—suggesting feedback to inform hippocampal disengagement and transition to a second stage. Notably, the first stage shows significant increases in hippocampal SWR–M1 slow oscillation coupling in the post-training sleep and is closely associated with rapid learning and variability of the M1 low-dimensional manifold. Strikingly, even after consolidation, inducing new manifold exploration by changing task parameters re-engages hippocampal–M1 coupling. We thus find evidence for dynamic hippocampal–cortical dialogue associated with manifold exploration during learning and adaptation.

## Abstract (web summary)

A new study explores the activity dialogue between hippocampus and cortex as neuronal representations of learning or adaptation are consolidated.

## Main

Memory stabilization through systems consolidation has long been hypothesized to follow a two-stage process^1–4^. Although both classic and recent studies provide support for a hippocampus-dependent and an independent phase^3,6,15,16^, it is unclear how cortical representations evolve with hippocampal–cortical dialogue. Moreover, it is unknown at what timescales such coordination occurs and what processes might inform transitions between stages during systems consolidation.

We use a prehension skill learning task to monitor hippocampal–cortical (prefrontal cortex (PFC) and primary motor cortex (M1)) dialogue across both sleep and awake states. Because prehension requires M1 (refs. ^14,17^), we can monitor consolidation in an essential region. Reactivation of task ensembles in M1 during non-rapid eye movement sleep (NREMS) is also known to be essential for skill learning^18–20^. Furthermore, global sleep slow oscillations (SOs), a phenomenon linked to the PFC and hippocampus^1,21–23^, appear to be critical drivers of local reactivation in M1 (ref. ^18^). Together, this suggests that consolidation of motor memories may involve dialogue between cortex and hippocampus during NREMS.

Although classic studies indicated that ‘procedural memories’ are hippocampus-independent^24^, recent functional MRI studies have found evidence for hippocampal activation in very early phases^25,26^. The nature of this hippocampal activation, however, remains unclear, and specifically whether hippocampal sharp-wave ripples (SWRs)—a key neurophysiological marker of memory consolidation during NREMS^1,27,28^—are associated with skill learning remains unknown. We therefore tested the hypothesis that time-varying changes in the coupling between SOs and SWRs during NREMS demarcate stages of consolidation.

A closely related hypothesis is that each of these two stages is associated with distinct phases of consolidation in M1. The temporal evolution of neural population dynamics (‘neural trajectories’) within a low-dimensional manifold (patterns of shared variance) is closely tied to skilled performance^29–31^. Whereas neural trajectories are stable for well-practised behaviours, they are variable during learning^32,33^. We quantified neural trajectory variance using a measure of fidelity. We expect low fidelity (large variance) during early learning because of ‘manifold exploration’. By contrast, during ‘manifold consolidation’, we expect increasing fidelity. It is unclear how systems consolidation might relate to manifold exploration versus consolidation.

Here, we demonstrate that changes in PFC–M1 SO coupling are closely linked to changes in performance across days and can both predict a drop in SWRs’ learning-related changes in M1 and demarcate a transition away from manifold exploration. Strikingly, there is a sharp increase in PFC–M1 SO coupling that coincides with stabilization of performance and manifold consolidation. This sharp increase in coupling is also a strong temporal predictor of the disengagement of increases in M1 SWR coupling; manifold consolidation continued to occur after this phase. Interestingly, inducing errors re-engaged manifold exploration and hippocampal–cortical coupling, further supporting that hippocampal–cortical dialogue may support manifold exploration.

## PFC–M1 SO coupling over long-term skill learning

**Fig. 1:**
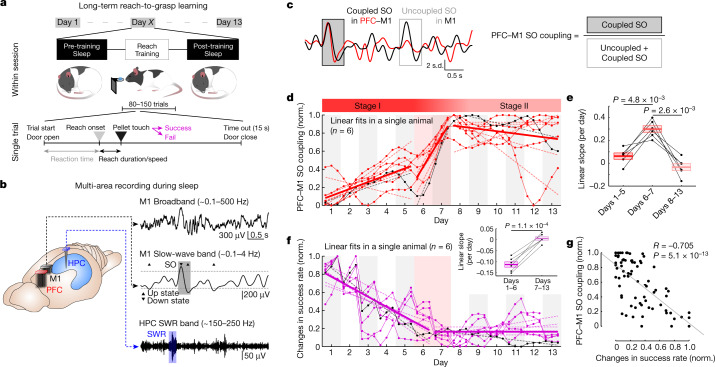
Changes in performance inversely correlated with increase in PFC–M1 SO coupling. **a**, Flow chart of reach-to-grasp task training experiment. **b**, Examples of the broadband (0.1–500 Hz) and the filtered local-field potential (LFP) trace in M1 for SOs (0.1–4 Hz) and in hippocampus (HPC) for SWRs (150–250 Hz) during sleep. SOs and SWRs are marked by grey and blue boxes, respectively. Horizontal dashed lines indicate the threshold detecting SOs up-/down-states and SWRs onset. **c**, Schematic showing measurement of temporal coupling of M1 SOs from PFC SOs, that is, PFC–M1 SO coupling. **d**, Time courses of PFC–M1 SO coupling. Lines represent piecewise linear regression fits. Piecewise linear regression fits are shown with dashed lines for the fits in a single animal (*n* = 6 animals) and with the solid line using all six animals. In each animal, scale of minimum-to-maximum was normalized to range from 0 to 1; same as **f** (details in Methods). **e**, Comparison of linear slopes across three periods (*n* = 6 animals); one-way analysis of variance (ANOVA), *F*_12,15_ = 33.39, *P* = 3.0 × 10^−6^; post hoc two-sided paired *t*-test, corrected for multiple comparison, days 1–5 versus days 6–7: *P* = 4.8 × 10^−3^, days 6–7 versus days 8–13: *P* = 2.6 × 10^−3^. Mean ± s.e.m. **f**, Time courses of changes in success rate (based on 2 d history; Extended Data Fig. 2e). Piecewise linear regression fits are shown with dashed lines for the fits in a single animal (*n* = 6 animals) and with the solid line using all six animals. Inset: linear slopes comparison (*n* = 6 animals); two-sided paired *t*-test, *t*_5_ = −10.93, *P* = 1.1 × 10^−4^. Mean ± s.e.m. **g**, Relationship between changes in success rate and PFC–M1 SO coupling. Across six rats, PFC–M1 SO coupling was well predicted by changes in success rate using a linear regression fit. Norm., normalized.

**Extended Data Fig. 1:**
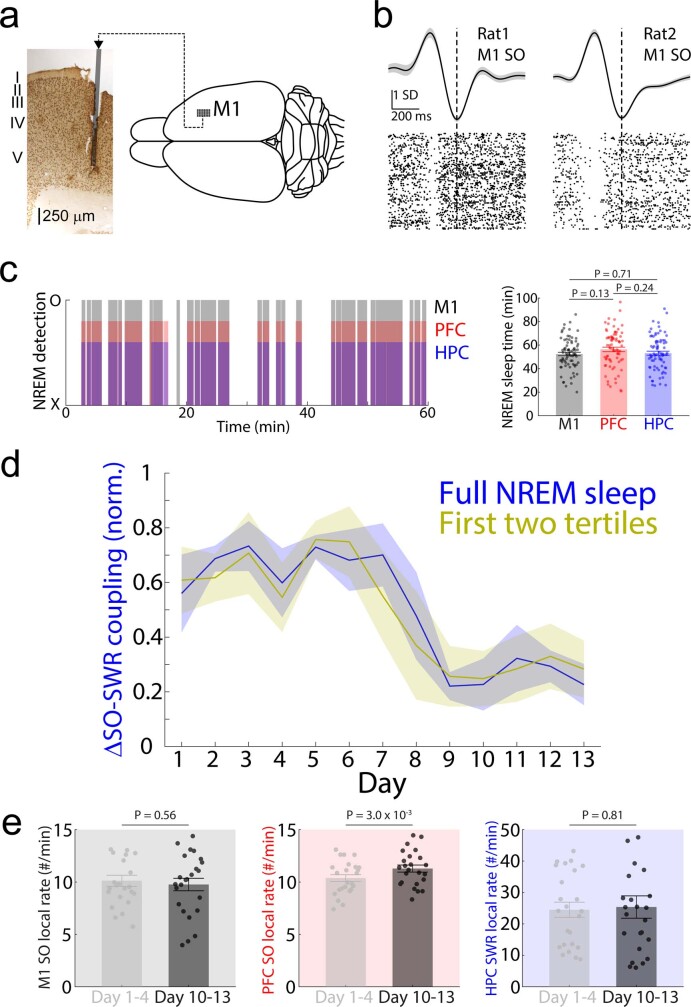
Validations of sleep and oscillation detections. **a**, Histology image of a coronal brain section showing the depth of a recording electrode (left) located at the middle of the microwire arrays in M1 (right). **b**, Average of SO-triggered LFP (top; 10-15 Hz), and raster plot of an example unit of two rats; locked to the SO up-states. Mean ± s.e.m. **c**, Examples of detections of NREMS using power spectral density in 0.1-4 Hz and gamma (30-60 Hz) bands independently using the LFP of M1 (black, n = 77 sessions in 6 rats), PFC (red, n = 77 sessions in 6 rats), and hippocampus (HPC, blue, n = 77 sessions in 6 rats). No significant difference in NREM sleep duration across three recording areas (one-way ANOVA, F_2,231_ = 1.47, P = 0.23; post hoc LME with two-sided *t*-test, corrected for multiple comparison, M1 vs. PFC: P = 0.13, M1 vs. HPC: P = 0.71, PFC vs. HPC: P = 0.24). Mean ± s.e.m. **d**, Average time course of ΔSO-SWR coupling (see Fig. 2) over the motor learning (n = 6 animals) using all detected NREM sleep (blue) versus using the first two tertiles (around early 66%) of the detected NREM sleep (yellow) in each sleep session. Mean ± s.e.m. **e**, Comparisons of rates between early period (n = 24 sessions in 6 rats) and late period (n = 24 sessions in 6 rats) in each corresponding metric (early days 1–4 vs. late days 10–13, LME with two-sided *t*-test; M1 SO: t_46_ = –0.60, P = 0.56; PFC SO: t_46_ = 3.13, P = 3.0 x 10^–3^; SWRs: t_46_ = 0.25, P = 0.81). Mean ± s.e.m.

**Extended Data Fig. 2:**
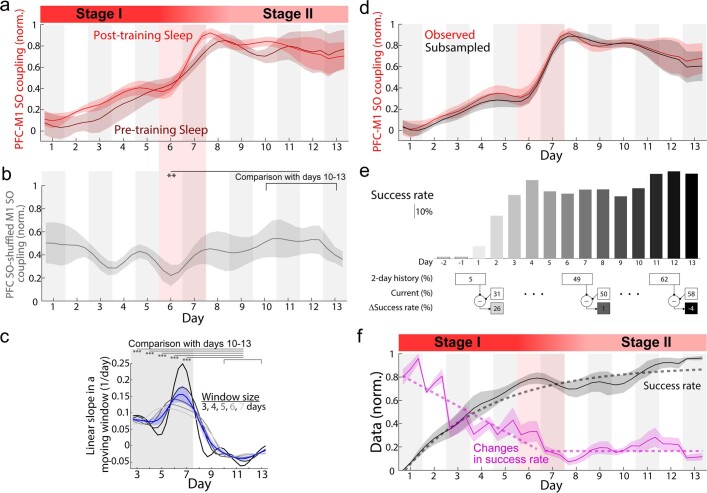
PFC-M1 SO coupling and changes in success rate. **a**, Average time courses of PFC-M1 SO coupling during post-training sleep (bright red) and pre-training sleep (dark red). The time course in pre-training sleep was normalized referencing to the post-training sleep in each animal. Mean ± s.e.m. **b**, Comparison of single-day PFC-M1 SO coupling with the mean during late period of days 10–13 in the M1-SO-shuffled condition. Top horizontal lines: significant difference from the days 10–13; one-way ANOVA, F_12,218_ = 1.73, P = 0.063; post hoc LME with two-sided *t*-test, corrected for multiple comparison, **P = 9.2 x 10^–3^. Mean ± s.e.m. **c**, Linear slopes of PFC-M1 SO coupling in a moving window. Gray-black traces represent measures with window sizes from 3-day (black) to 7-day (lightest gray). Blue represents average trace across the window sizes. Horizontal lines: significant difference from the days 10–13 using the 3-day size, LME with two-sided *t*-test, corrected for multiple comparison, ***P < 6.8 x 10^–9^. Mean ± s.e.m. **d**, Computation of the PFC-M1 SO coupling during post-training sleep after subsampling PFC SOs to make the number of SOs used in the computation match across sessions (Methods). No session showed a significant difference between the observed condition for all PFC SOs and the subsampled condition; two-sided paired *t*-test, P > 0.28 for all sessions. Mean ± s.e.m. **e**, Computation of the changes in success rate (ΔSuccess rate) from the two-day history; it is shown using the task performance of an example animal. **f**, Pellet retrieval success rates (black) and changes in success rate (magenta) in average across animals (n = 6 rats). Black dashed curve indicates single-exponential function fit (R^2^ = 0.85, P = 3.9 x 10^–11^). Magenta dashed lines indicate piecewise linear regression fits. Mean ± s.e.m.

If cortical interactions during NREMS play a role in consolidation, then there should be changes in the coordination of SOs in PFC and M1 with learning. We measured performance in a reach-to-grasp task over approximately 13 d (*n* = 6 rats; Fig. 1a), while also monitoring SOs in M1 and PFC as well as hippocampal SWRs (Fig. 1b and Extended Data Fig. 1). We first examined changes in the precise temporal coupling between SOs in PFC and M1. ‘PFC–M1 SO coupling’ is the number of M1 SOs occurring within 200 ms from a PFC SO divided by the total number of SOs in M1 (Fig. 1c). We examined changes in PFC–M1 SO coupling within and across sessions, particularly as there may be slow changes over days^33,34^. Indeed, PFC–M1 SO coupling demonstrated slow changes across days, but values in ‘pre-training’ and the ‘post-training’ sleep were similar within a day (Extended Data Fig. 2a). We thus focused only on the post-training sleep to examine PFC–M1 SO coupling over long-term learning. Notably, we found significantly higher PFC–M1 SO coupling in the late compared with the early period (Fig. 1d; normalization based on the minimum and maximum values across all days; ‘early’ is days 1–4 and ‘late’ is days 10–13).

We next examined the temporal dynamics of the PFC–M1 SO coupling. We used moving windows of varying lengths (3–7 d) to identify rates of change and the location of rapid transitions (Methods and Extended Data Fig. 2c). We found a sharp transition around day 6 in all animals. On this basis, we measured linear slopes for the three periods and found a significantly fast transition on days 6–7 (Fig. 1e). After this period, there was elevated PFC–M1 SO coupling. There was no significant change in the rates of M1 SOs (Extended Data Fig. 1e). Although there was a significant increase in the rates of PFC SOs, this could not account for changes in coupling. First, when we generally shuffled the timing of PFC and M1 SOs, we did not observe this change (Extended Data Fig. 2b). Second, when we ‘subsampled’ the number of PFC SOs to match rates across sessions, we still observed nearly identical increases in coupling (Extended Data Fig. 2d). Finally, total recorded NREMS periods were similar in both early and late (days 1–4, *n* = 24 sessions in 6 rats: 53.35 ± 2.34 min versus days 10–13, *n* = 24 sessions in 6 rats: 49.03 ± 2.29 min, linear mixed-effects model (LME) with two-sided *t*-test, *t*_46_ = −1.61, *P* = 0.11). Thus, there was more precise coupling of PFC–M1 SOs with learning.

The transition from low to high PFC–M1 SO coupling occurred as the success rate stabilized (Fig. 1f,g; example shown in Extended Data Fig. 2e,f). To examine the rates of performance change before and after the transition day, we fit a line to the changes in success rate for each animal for days 1–6 and days 7–13. This revealed an initial reduction in the change of success rate (behavioural exploration); this was followed by stable performance. This indicates that the transition from exploration to stable performance was temporally linked to a sharp increase in PFC–M1 SO coupling.

## Stepwise drop in M1 SO–SWR coupling

**Fig. 2:**
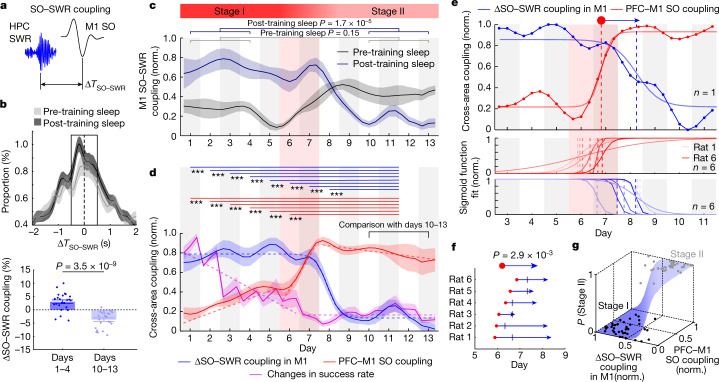
Sharp increase in PFC–M1 SO coupling predicts stepwise drop in SWR–M1 SO coupling. **a**, SO–SWR coupling in M1: temporal lags of SWRs from M1 SO, Δ*T*_SO–SWR_. **b**, Comparison of M1 SO–SWR coupling (box: probability of SWR in −0.75 to +0.75 s window relative to SO). Top, Δ*T*_SO–SWR_ distribution during pre- (*n* = 24 sessions in 6 rats, also hereafter) and post-training sleep of early. SO–SWR coupling in window was higher in post-training sleep (two-sided paired *t*-test, *t*_23_ = −2.84, *P* = 9.3 × 10^−3^). Bottom, ΔSO–SWR coupling change of SO–SWR (pre- versus post sleep) higher during early (days 1–4) versus late (days 10–13); LME, two-sided *t*-test, *t*_46_ = −7.28, *P* = 3.5 × 10^−9^. Mean ± s.e.m., also hereafter. **c**, Average time course of M1 SO–SWR coupling over the motor learning during pre-training sleep (early: 22.71 ± 0.98 versus late: 24.35 ± 1.05, LME, two-sided *t*-test, *t*_46_ = 1.61, *P* = 0.15) and post-training sleep (early: 24.95 ± 0.85 versus late: 20.47 ± 0.83, LME, two-sided *t*-test, *t*_46_ = −4.81, *P* = 1.7 × 10^−5^). The time course in pre-training sleep was normalized, referencing to the post-training sleep in each animal. **d**, Average time course of PFC–M1 SO coupling (red), ΔSO–SWR coupling in M1 (blue) and changes in success rate (magenta). Thick dashed sigmoid curve indicates sigmoid function fit. Thick dashed linear lines indicate piecewise linear regression fits reproduced from Fig. 1d,f. Top horizontal lines: significant difference from the days 10–13; PFC–M1 SO coupling: one-way ANOVA, *F*_12,218_ = 44.66, *P* = 5.0 × 10^−52^; post hoc LME, two-sided *t*-test, corrected for multiple comparison, ***P < 5.2 × 10^−5^; ΔSO–SWR coupling: one-way ANOVA, *F*_12,218_ = 29.30, *P* = 4.6 × 10^−39^; post hoc LME, two-sided *t*-test, corrected for multiple comparison, ***P < 2.2 × 10^−6^. **e**,**f**, Comparison of stepwise increase of PFC–M1 SO coupling with drop in ΔSO–SWR coupling in single animal (**e**, top); middle and bottom, sigmoid function fits for each animal. Sharpest changes marked by red circles and blue arrowheads. Significant for six animals; two-sided paired *t*-test, *P* = 2.9 × 10^−3^ (**f**); black bars represent stage transitions using change-point analysis. **g**, Probability of stage II predicted using logistic regression as function of ΔSO–SWR coupling in M1 and PFC–M1 SO coupling (blue surface). ‘Stage I’ (black) and ‘Stage II’ (grey) represent true sessions before and after grand-midpoint (median of red dot and blue arrowhead in an individual animal in **f**).

**Extended Data Fig. 3:**
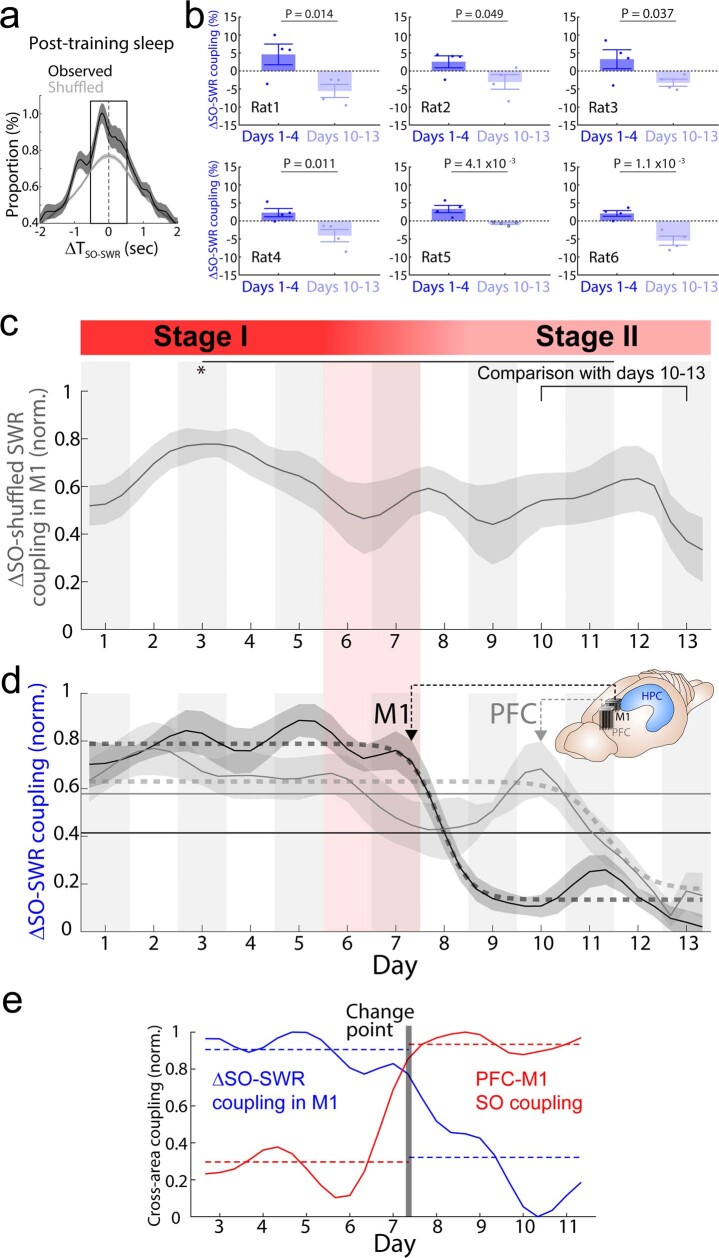
Comparis×ons in ΔSO-SWR coupling. **a**, Comparison of distributions of ΔT_SO-SWR_ in M1 during post-training sleep in early learning (days 1–4). The SO-SWR coupling (probability of SWRs −0.75 to +0.75 sec from an SO, black box) during post-training sleep was compared with the SWR-shuffled condition (two-sided paired *t*-test, t_23_ = –0.34, P = 0.017). Mean ± s.e.m. **b**, Comparison of M1 ΔSO-SWR coupling during early (days 1—4) versus late period (days 10–13) in an individual animal. Mean of absolute M1 ΔSO-SWR coupling was significantly lower during the late compared to the early period; LME with two-sided *t*-test, P = 0.014, 0.049, 0.037, 0.011, 4.1 × 10^–3^, and 1.1 × 10^–3^ for Rats 1–6. Mean ± s.e.m. **c**, Comparison of single-day M1 ΔSO-SWR coupling with the mean during late period of days 10—13 in the SWR-shuffled condition (i.e., temporal coupling was measured using the intact SOs and temporally disrupted structure of SWRs); one-way ANOVA, F_12,218_ = 3.10, P = 4.5 × 10^–4^; post hoc LME with two-sided *t*-test, corrected for multiple comparison, *P = 0.016. Mean ± s.e.m. **d**, Average time course of ΔSO-SWR coupling between PFC and hippocampus over the motor learning (gray; n = 6 rats); black is reproduced for M1 in Fig. 2d. Thick dashed curves indicate sigmoid function fits. Horizontal lines indicate the mean level during the two days prior to day 1. Mean ± s.e.m. **e**, Stage change point estimation using ‘change-point analysis’ for the single animal example in Fig. 2e. Black bars represent the estimated time point of stage transition. Dashed lines represent mean at each stage.

We next wondered whether the increased PFC–M1 coordination is temporally associated with changes in hippocampus–M1 coordination, as would be predicted for a memory consolidation process in M1 that evolves to become hippocampus-independent^1–4,35^. We first quantified coordination between hippocampal SWRs (Fig. 1b) and M1 SOs by measuring the time lag between an M1 SO and its nearest SWR (Δ*T*_SO–SWR_; Fig. 2a). We measured ‘SO–SWR coupling’ (probability of SWRs −0.75 to +0.75 s from an SO; black box in Fig. 2b and Methods) in the pre- and post-training sleep. In comparison with both a pre-training sleep and a shuffled distribution, the post-training sleep in early learning (days 1–4) had a significantly greater incidence of SWRs with precise temporal proximity to M1 SOs (Fig. 2b and Extended Data Fig. 3a). Notably, whereas PFC–M1 SO coupling demonstrated a slow change over days, SO–SWR coupling showed a faster learning-related change, that is, strong changes from pre- to post-training sleep (Fig. 2c). These results are perhaps consistent with views of the hippocampus and the cortex being, respectively, fast and slow learning systems^2^.

To quantify changes in hippocampal–M1 coordination, we measured ‘ΔSO–SWR coupling’ in M1 as the normalized difference of coupling between post- and pre-training sleep. There was significantly stronger ΔSO–SWR coupling during the early versus the late period (Fig. 2b and Extended Data Fig. 3b). The temporal dynamics of ΔSO–SWR coupling in M1 also showed stepwise dynamics (Fig. 2d, sigmoid function fit, *R*^2^ = 0.97, *P* = 1.6 × 10^−46^). There was a marked drop slightly after the sharp increase in PFC–M1 SO coupling (days 6–7). This was not the case if we disrupted the SWR temporal structure by shuffling across time (Extended Data Fig. 3c). This was also not due to a difference in SWR rates (Extended Data Fig. 1e). Notably, the ΔSO–SWR coupling in PFC showed sustained higher values and a delayed drop (Extended Data Fig. 3d).

To examine temporal links between cross-area coupling transitions, we fit a sigmoid function to each animal’s cross-area coupling (thick curves in Fig. 2e and Methods). Across six rats, the stepwise dynamics were well predicted by a sigmoid function in ΔSO–SWR coupling in M1 (fit per animal, *R*^2^ = 0.66 ± 0.078, *P* < 3.27 × 10^−4^) and in PFC–M1 SO coupling (fit per animal, *R*^2^ = 0.86 ± 0.056, *P* < 4.8 × 10^−19^). This increase in PFC–M1 SO coupling—as measured by the midpoint of the sigmoid function fit, that is, the red dot—was followed by a sharp drop in ΔSO–SWR coupling in M1 (blue arrowhead). This lag was significant across the six animals (Fig. 2f; rise time in PFC–M1 SO coupling, that is, red dots, *n* = 6 rats: 6.2 ± 0.16 d versus drop time in ΔSO–SWR coupling in M1, that is, blue arrowheads, *n* = 6 rats: 7.9 ± 0.23 d; two-sided paired *t*-test, *t*_5_ = −5.4, *P* = 2.9 × 10^−3^). We arrived at a very similar conclusion when estimating the transition using ‘change-point analysis’ (black bars in Fig. 2f, Extended Data Fig. 3e and Methods).

These findings indicate that the full engagement of NREMS PFC–M1 coordination predicts hippocampal–M1 disengagement. The median of the rise time in PFC–M1 coordination and the drop time in hippocampal–M1 coordination (hereafter, grand-midpoint) suggest two distinct stages of consolidation, hereafter termed stage I or stage II. We thus assessed whether knowledge about hippocampus, PFC and M1 states could identify whether a given animal was in stage I or stage II. We quantified this using a logistic regression model of ΔSO–SWR coupling in M1 and PFC–M1 SO coupling to predict whether a given session was stage I or II. The probability of stage II was significant using this model (*P* = 4.3 × 10^−3^ for ΔSO–SWR coupling in M1 and *P* = 2.4 × 10^−3^ for PFC–M1 SO coupling; Fig. 2g). Thus, our results indicate that changes in PFC–M1 coordination and hippocampal–M1 disengagement can define two distinct phases during systems consolidation.

**Extended Data Fig. 4:**
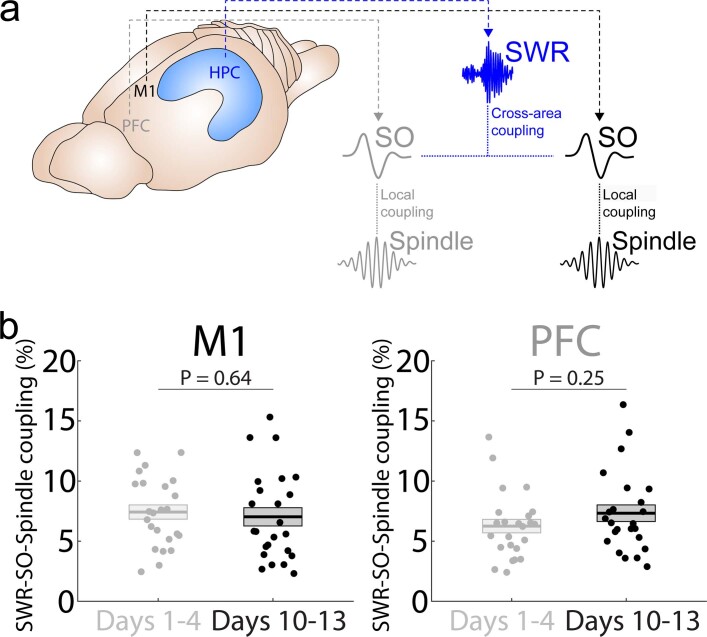
No change in cross-area coupling of spindles over learning. **a**, Schematic model of the relationship across SWRs, SOs, and spindles. SOs are coupled across PFC and M1 in relation to SWRs. These coordinated SOs also demonstrate increased communication across PFC-M1 (Fig. 4). Spindles are coupled to SOs in a local area processing but are not common during a cross-area coupled SO period. Our data suggest that the cross-area communication is related to SO-coupling and orchestration by SWR, while the spindle activity appears to be modulated by an area-specific process (e.g., corticothalamic connections projecting from each cortical area). **b**, Comparisons of the mean of triple phase-coupling (i.e., precise coupling of SWR, SO and spindle) in M1 (early days 1—4, n = 24 sessions in 6 rats: 7.4 ± 0.59% vs. late days 10–13, n = 24 sessions in 6 rats: 7.0 ± 0.75%, LME with two-sided *t*-test, t_46_ = –0.47, P = 0.64) and PFC (early days 1—4, n = 24 sessions in 6 rats: 6.3 ± 0.56% vs. late days 10–13, n = 24 sessions in 6 rats: 7.3 ± 0.69%, LME with two-sided *t*-test, t_46_ = 1.23, P = 0.25). Mean ± s.e.m.

**Extended Data Fig. 5:**
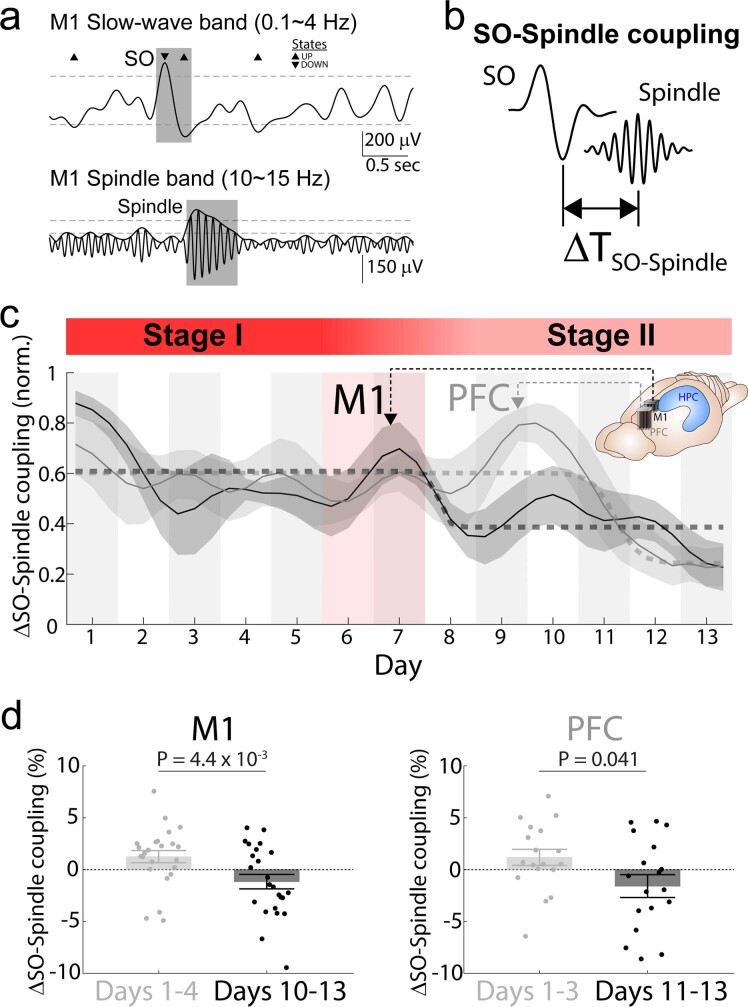
Changes in spindles coupling with SO over learning. **a**, Examples of the filtered LFP trace in M1 for SOs (0.1-4 Hz; same trace as in Fig. 1b) and spindles (10-15 Hz) during NREM sleep. SO and spindle are marked by gray boxes. Horizontal dashed lines indicate the threshold detecting SOs up/down states, and spindles onsets (lower dashed line) or high spindle power (upper dashed line). **b**, Schematic of SO-Spindle coupling, measured as the temporal lags of spindles from a SO up-state, ΔT_SO-Spindle_. SO-Spindle coupling was measured by coupling probability focused on the spindles from –0.5 to +1.0 sec from the time of the SO up-state (Methods). The difference of coupling between post-training sleep and pre-training sleep was examined over learning in ‘**c**’ (ΔSO-Spindle coupling). **c**, Average time course of ΔSO-Spindle coupling in PFC (gray) and M1 (black) over the motor learning (n = 6 rats). Thick dashed curves indicate sigmoid function fits. Mean ± s.e.m. **d**, Comparisons of the mean of ΔSO-Spindle coupling in M1 (early days 1—4, n = 24 sessions in 6 rats: 1.3 ± 0.82% vs. late days 10–13, n = 24 sessions in 6 rats: –1.2 ± 0.62%, LME with two-sided *t*-test, t_46_ = –2.99, P = 4.4 × 10^–3^) and PFC (early days 1—3, n = 24 sessions in 6 rats: 1.18 ± 0.79% vs. late days 11–13, n = 24 sessions in 6 rats: –1.59 ± 0.98%, LME with two-sided *t*-test, t_46_ = –2.12, P = 0.041). Mean ± s.e.m.

Memory consolidation is known to require the precise coupling of sleep spindles to SOs and SWRs^18,36,37^. We examined the temporal relationship of spindles to SOs and SWRs. Our analyses suggest that whereas cross-area communication is related to SOs (possible orchestration by SWRs), spindle activity appears to be a local process (model illustrated in Extended Data Fig. 4a). We did not observe changes in ‘triple-coupling’ (that is, precise coupling of SWR, SO and spindle; Extended Data Fig. 4b). However, we found learning-related changes in the local coupling of spindles with M1 SOs and PFC SOs separately (Extended Data Fig. 5). There were stronger task-dependent changes in the SO–Spindle coupling during the early versus the late period (that is, pre- versus post-training sleep, ΔSO–Spindle coupling^18,38^). This suggest a model in which changes in area-specific SO–Spindle coupling are important. However, spindles do not appear to demonstrate a specific change over days during coordinated SWR and SO events.

## Manifold consolidation with drop in SO–SWR coupling

**Fig. 3:**
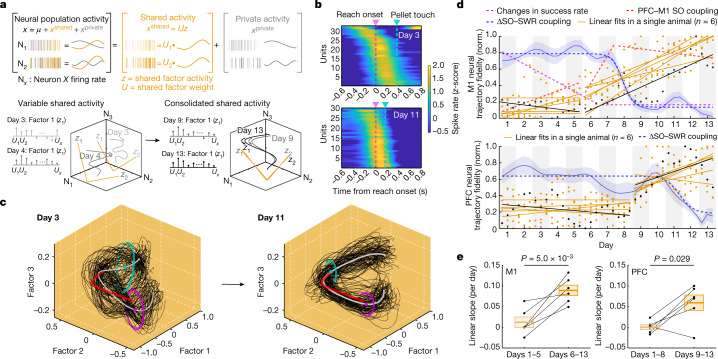
Changes in the fidelity of population dynamics. **a**, Schematic diagram showing shared and private activity of neural population activity using GPFA. **b**, Examples of PETH in M1 during reach period at days 3 and 11; locked to the reach onsets. Durations from reach onset to pellet touch were typically longer at the early compared with the late period of learning. **c**, Examples of neural trajectories (black) travelling on the shared activity space defined by the top three GPFA factors in M1. Grey curves, mean template. Red curves, ‘optimal’ neural trajectory for a successful task. Dots, mean reach onset (magenta) and pellet touch (cyan) times. Ellipses, 90% confidence area of covariance. **d**, Neural trajectory fidelity over learning in M1 (top) and PFC (bottom); each dot represents measurement in a tertile of single-session trials. Lines represent linear regression fits in each animal for either days 1–5 or days 6–13 in M1 and days 1–8 or days 9–13 in PFC. Top: red and magenta dashed lines represent the piecewise linear regression fits of the changes in PFC–M1 SO coupling from Fig. 1d and in success rate from Fig. 1f, respectively. Blue curves represent ΔSO–SWR coupling from Fig. 2d and Extended Data Fig. 3d. **e**, Comparison of the slopes in **d** between two periods (two-sided paired *t*-test, *n* = 6 rats, *P* = 5.0 × 10^−3^ for M1, *P* = 0.029 for PFC). Transition period was determined by varying windows (Extended Data Fig. 6e). Mean ± s.e.m.

**Extended Data Fig. 6:**
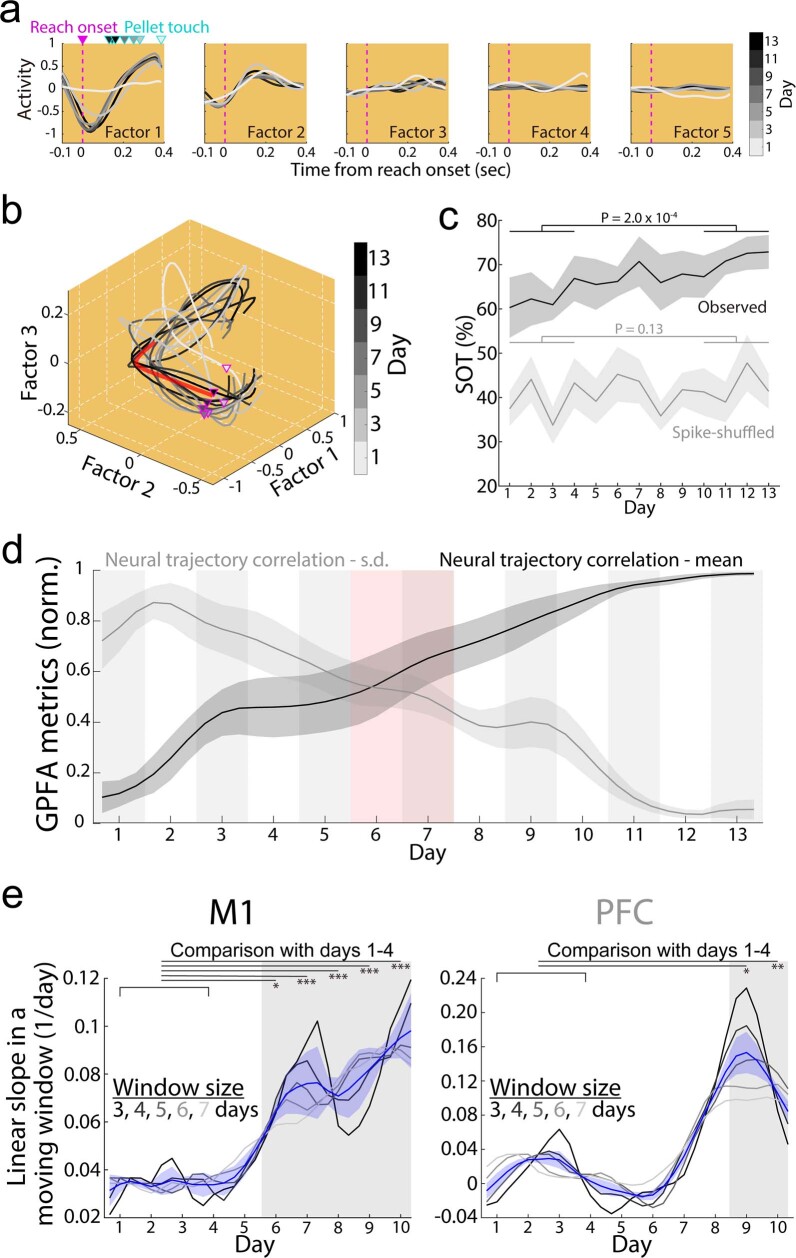
Examples of neural trajectory templates and metrics of GPFA. **A**, Examples of M1 neural trajectories traveling on the five GPFA factors in an animal. Gray curves with gradient intensity: mean templates of each session noted on the color bar. Triangles: reach onset (magenta) and mean pellet touch (cyan outline with filled by gray colors) time. **b**, Examples of mean neural trajectories traveling on the shared activity space defined by the top 3 GPFA factors in M1. Red curves: optimal neural trajectory for a successful task. Magenta triangles: reach onsets of each session. **c**, Average trace of shared-over-total variance (SOT) in M1 using all GPFA factors in the observed condition (black; early days 1–4, n = 24 sessions in 6 rats: 62.5 ± 2.6% vs. late days 10–13, n = 24 sessions in 6 rats: 71.4 ± 1.9%, LME with two-sided *t*-test, t_46_ = 4.03, P = 2.0 × 10^–4^) and the spike-shuffled condition (gray; early days 1–4, n = 24 sessions in 6 rats: 38.8 ± 2.4% vs. late days 10–13, n = 24 sessions in 6 rats: 42.6 ± 2.5 %, LME with two-sided *t*-test, t_46_ = 1.54, P = 0.13). n.s.: non-significance. Mean ± s.e.m. **d**, Average trace across animals for the mean (black) and the standard-deviation (s.d.; gray) of neural trajectory correlations in M1 (n = 6 rats). Mean ± s.e.m. **e**, Linear slopes of mean neural trajectories in a moving window in M1 and PFC. Gray-black traces represent measures with window sizes from 3-day (black) to 7-day (lightest gray). Blue represents average trace across the window sizes. Horizontal lines: significant difference from the days 1—4 using the 5-day size, LME with two-sided *t*-test, corrected for multiple comparison, *P = 0.032, ***P < 7.6 × 10^–4^ for M1, *P = 0.011, **P = 3.2 × 10^–3^ for PFC; results were similar with the other window size. Mean ± s.e.m.

We investigated how these cross-area interactions relate to changes in awake task-related activity during learning. On the basis of a model that early skill learning is associated with manifold exploration and later learning is associated with the stabilization of population dynamics (manifold consolidation)^29,32,39,40^, we monitored the stability of population dynamics in M1 and PFC using Gaussian process factor analysis (GPFA). GPFA identifies a low-dimensional cortical manifold which represents patterns of neural co-firing (that is, shared activity; Fig. 3a)^29,32,33^. We calculated GFPA neural trajectories for each day and focused on the top three factors during reaching (Fig. 3b; see Methods for alignment across days). First, we focused on M1. As expected, neural trajectories were variable across trials in a representative early session, whereas they were more stereotyped in a late session (Fig. 3c and Extended Data Fig. 6a–d). Consistency across days, particularly for periods of stable performance, is also consistent with recent findings of stable manifolds across days^32,41^.

We next quantified the changes in trajectory stability over days. To do so, we calculated a ‘GPFA correlation (*R*)’, defined as the correlation coefficient of an individual neural trajectory with the average neural trajectory of the final 3 d associated with the best performance (Methods). We then calculated a neural trajectory ‘fidelity’, a signal-to-noise ratio^42–44^ of GPFA *R* values across days; it captured the consistency of GPFA *R* values across trials. The dots (orange for *n* = 5 and black for a single animal) in Fig. 3d show trends towards increased fidelity.

On the basis of the observed changes in PFC–M1 SO coupling (Fig. 1d), we wondered whether there might be two distinct stages for activity patterns within the M1 manifold. We again used moving windows (for example, 3–7 d) to estimate slopes to objectively determine the greatest change in M1 fidelity; we found that between days 5 and 6 was a consistent transition point (Extended Data Fig. 6e). This suggested that days 1–5 and days 6–13 represented two periods with distinct rates of change. We thus fit a line for each animal for days 1–5 and then days 6–13. This demarcation revealed an essentially flat slope of approximately zero for days 1–5 (manifold exploration) and then a gradual linear increase trajectory fidelity (manifold consolidation, Fig. 3d,e). Notably, the period of highly variable success rate (Fig. 1f) also appears to be closely related to this transition. Together, these findings raise the possibility that the transition from exploration—associated with variable performance and a low fidelity—to consolidation is temporally linked to changes in PFC–M1 coupling.

We then repeated the same analyses for PFC (Fig. 3d,e). We found that manifold consolidation in PFC occurred in a delayed fashion relative to M1; this appears to be closely linked to the delayed disengagement of PFC SO–SWR coordination. Thus, changes in ΔSO–SWR coupling appear to follow the onset of neural trajectory stabilization in each area, PFC and M1.

## SWRs predict increased PFC–M1 communication

**Fig. 4:**
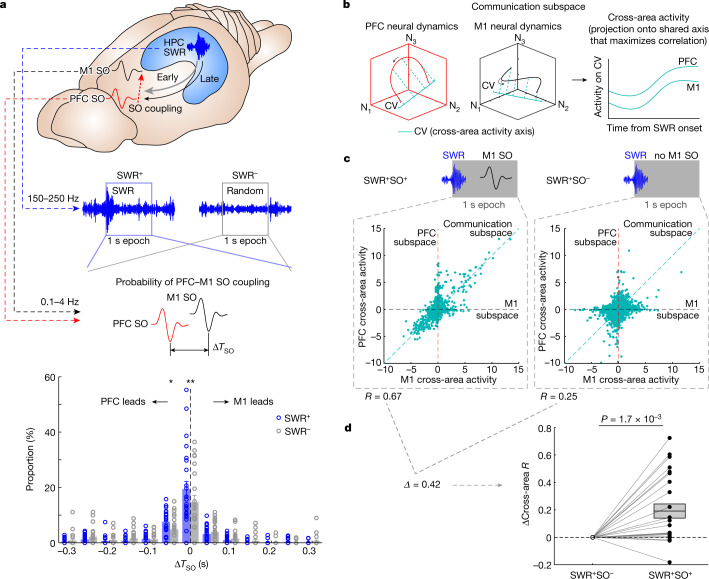
Higher PFC–M1 communication is associated with SWR-linked events. **a**, Comparison in distributions of PFC SOs from M1 SOs (Δ*T*_SO_) between the SWR^+^ (1 s epochs following SWRs onsets) and the SWR^−^ condition (randomly selected 1 s NREMS epochs) in the early period (days 1–4). *P = 0.012 and **P = 7.0 × 10^−3^: significant difference in each 50 ms bin between two conditions using two-sided paired *t*-test (*n* = 24 sessions in 6 rats). Mean ± s.e.m. **b**, Schematic diagram showing the communication subspace (cross-area activity axes/canonical variable (CV)) in PFC and M1. Neural dynamics (three neurons’ spike activity, N_1–3_) in each area are projected onto the respective CV that maximizes correlation between two areas, that is, cross-area activity showing correlated time course between PFC and M1. **c**,**d**, PFC and M1 cross-area activities during the SWR^+^SO^+^ and the SWR^+^SO^−^ in the early period of days 1–4 (**c**). There was stronger correlation between PFC and M1 cross-area activities during the SWR^+^SO^+^ compared with the SWR^+^SO^−^, which is shown as the difference between the two conditions (ΔCross-area *R* in **d**; *n* = 24 sessions in 6 rats, two-sided paired *t*-test, *P* = 1.7 × 10^−3^). Mean ± s.e.m.

**Extended Data Fig. 7:**
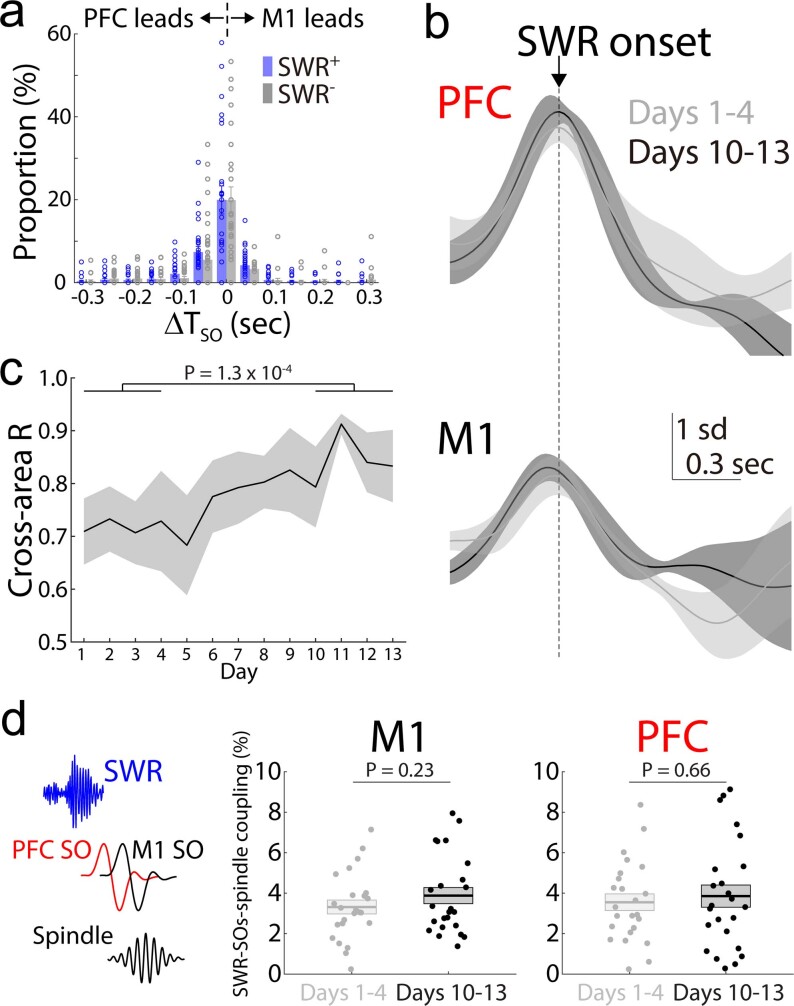
Cross-area communications during long-term motor learning. **a**, Comparison of distributions of PFC SOs from M1 SOs (ΔT_SO_) during the late period of days 10–13 between the SWR^+^ (1-sec epochs following SWRs onsets) and SWR^-^ condition (randomly selected 1-sec NREM epochs). No significant difference was found in each 50 ms bin between two conditions using two-sided two-sampled *t*-test (n = 24 sessions in 6 rats). Mean ± s.e.m. **b**, Average of SWR-onset-triggered PETH in PFC and M1 (early days 1–4: n = 24 sessions in 6 rats; late days 10—13: n = 24 sessions in 6 rats). Mean ± s.e.m. **c**, Average trace of the correlation between PFC and M1 cross-area activities (Cross-area R) over the motor learning in the SWR^+^ condition (early days 1—4, n = 24 sessions in 6 rats: 0.72 ± 0.033 vs. late days 10–13, n = 24 sessions in 6 rats: 0.84 ± 0.029, LME with two-sided *t*-test, t_46_ = 3.41, P = 1.3 × 10^–4^). Mean ± s.e.m. **d**, Comparisons of the percentage of quadruple phase-coupling (i.e., precise coupling of SWR, PFC SO, M1 SO, and spindle). There was no significant difference between the early versus the late period in M1 (early days 1—4, n = 24 sessions in 6 rats: 3.32 ± 0.34% vs. late days 10—13, n = 24 sessions in 6 rats: 3.87 ± 0.40%, LME with two-sided *t*-test, t_46_ = 1.22, P = 0.23) and PFC (early days 1—4, n = 24 sessions in 6 rats: 3.55 ± 0.41% vs. late days 10—13, n = 24 sessions in 6 rats: 3.85 ± 0.55%, LME with two-sided *t*-test, t_46_ = 0.45, P = 0.66). Mean ± s.e.m.

The current model of systems consolidation posits that hippocampal SWRs serve to enhance coordination across cortical networks^9,11,21^. We, therefore, asked whether the presence of SWRs predicts increased cross-area communications between PFC and M1. We focused on the probability of PFC–M1 SO coupling during SWRs (Fig. 4a). We measured the temporal lags between M1 SOs up-states and the nearest PFC SOs up-states (Δ*T*_SO_). During early sessions (days 1–4), we found that the distribution of Δ*T*_SO_ indicated stronger PFC–M1 SO coupling during a 1 s epoch following an SWR (SWR^+^) compared with a randomly selected 1 s epoch of NREMS (SWR^−^) (no significant difference in the distributions during the late sessions; Extended Data Fig. 7a).

Next, we quantified changes in ‘communication subspace’^45^ dynamics between PFC and M1 using canonical correlation analysis (CCA)^40^ (Fig. 4b). The correlation values of projected data allow us to assess whether there is increased correlated activity across the areas. Cross-area *R* between PFC and M1 was measured during 1 s periods after SWRs (spike peri-event time histogram (PETH) in Extended Data Fig. 7b), and it showed learning-related increases (Extended Data Fig. 7c). Furthermore, cross-area *R* was examined for either SWRs with an SO within 1 s (SWR^+^SO^+^) or SWRs without a following SO (SWR^+^SO^−^) (Fig. 4c). The mean cross-area *R* was significantly higher during the SWR^+^SO^+^ compared with the SWR^+^SO^−^ (SWR^+^SO^+^, *n* = 24 sessions in 6 rats: 0.46 ± 0.061 versus SWR^+^SO^−^, *n* = 24 sessions in 6 rats: 0.26 ± 0.036, two-sided paired *t*-test, *t*_23_ = −3.72, *P* = 1.7 × 10^−3^; shown as ΔCross-area *R* in Fig. 4d). Notably, these changes in cross-area communication during SWR^+^ and SWR^+^SO^+^ were not associated with changes in coupling to spindles (Extended Data Fig. 7d). These analyses support the notion that SWRs are linked to significantly elevated PFC–M1 cross-area communication during SO coupling.

## M1 reactivation of population dynamics during SWRs

**Fig. 5:**
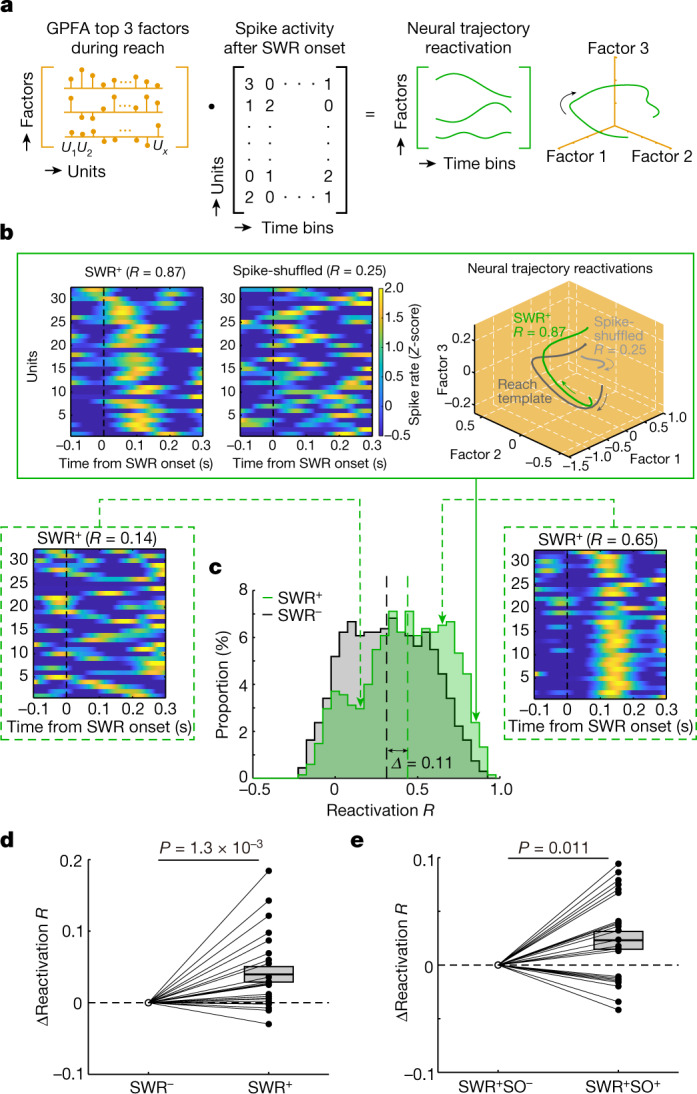
SWRs with a following SO enhance motor memory reactivations in M1. **a**, Schematic diagram showing computation of M1 neural trajectory reactivations. Neural ensemble activity during the SWRs period was projected onto the top three GPFA factors defined during the awake experience in Fig. 3; that is, defined as a neural trajectory reactivation. See Methods for the details. **b**, Three examples of neural trajectory reactivations, that is, spike ensemble activity during an SWR. The SWR^+^ showed higher correlation to reach the template compared with the SWR-shuffled condition (box with solid line); reactivation *R* was measured comparing a neural trajectory reactivation during sleep with the reach template during task. **c**, Distributions of reactivation *R* values in an example session. We focused on the difference in the mean of reactivation *R* values (vertical dashed lines) between the two comparing conditions (*Δ* = the value in SWR^+^ − the value in SWR.ind.; that is, ‘ΔReactivation *R*’ in **d** and **e**). Significant difference in two distributions (two-sided two-sample Kolmogorov–Smirnov test, *k* = 0.19, *P* = 4.4 × 10^−4^). **d**, Comparisons of the mean of reactivation *R* values between the SWR^+^ versus the SWR^−^ condition (*n* = 24 sessions in 6 rats, two-sided paired *t*-test, *P* = 1.3 × 10^−3^). Shown is the difference between the two conditions (ΔReactivation *R*; same as **e**). Mean ± s.e.m. **e**, Comparisons of the mean of reactivation *R* values between the SWR^+^SO^+^ versus the SWR^+^SO^−^ (*n* = 24 sessions in 6 rats, two-sided paired *t*-test, *P* = 0.011). Mean ± s.e.m.

**Extended Data Fig. 8:**
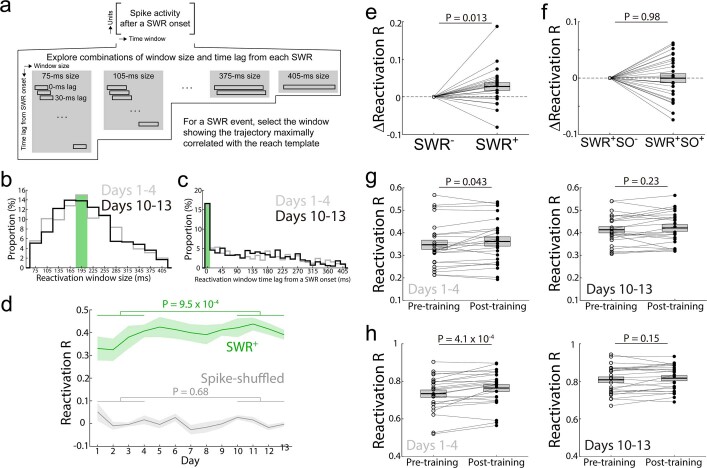
Parameters and changes over motor learning in M1 neural trajectory reactivations. **a**, Schematic for finding optimal window size and time lag for the neural trajectory reactivation after a SWR onset. See the details in Methods for determining optimal window size. **b—c**, the distributions of window sizes and time lags of the final neural trajectory reactivations for the early period (899 SWRs during days 1—4, n = 6 rats) and the late period (826 SWRs during days 10–13, n = 6 rats). **d**, Average trace of reactivation correlation (R) over long-term motor learning (n = 6 rats) in the SWR^+^ condition (early days 1–4, n = 24 sessions in 6 rats: 0.36 ± 0.023 vs. late days 10–13, n = 24 sessions in 6 rats: 0.42 ± 0.015, LME with two-sided *t*-test, t_46_ = 3.53, P = 9.5 × 10^–4^) versus the spike shuffled condition (early days 1–4, n = 24 sessions in 6 rats: 0.014 ± 0.012 vs. late days 10–13, n = 24 sessions in 6 rats: 0.0064 ± 0.0072, LME with two-sided *t*-test, t_46_ = –0.42, P = 0.68). n.s.: non-significance. Mean ± s.e.m. **e**, Comparisons of the mean of reactivation Rs between the SWR^+^ versus the SWR^−^ condition during the late period of days 10–13 (n = 24 sessions in 6 rats, SWR^+^: 0.39 ± 0.017 vs. SWR^−^: 0.42 ± 0.015, two-sided paired *t*-test, t_23_ = –2.69, P = 0.013). Mean ± s.e.m. **f**, Comparisons of the mean of reactivation Rs between the SWR^+^SO^+^ versus the SWR^+^SO^−^ during the late period of days 10–13 (n = 24 sessions in 6 rats, SWR^+^SO^+^: 0.39 ± 0.018 vs. SWR^+^SO^−^: 0.39 ± 0.020, two-sided paired *t*-test, t_23_ = 0.025, P = 0.98). n.s.: non-significance. Mean ± s.e.m. **g**, Comparisons of the mean of all reactivation Rs in SWR^+^ between pre- versus post-training sleep during the early (days 1–4, left; n = 24 sessions in 6 rats, pre-training: 0.35 ± 0.021 vs. post-training: 0.38 ± 0.020, two-sided paired *t*-test, t_23_ = –2.14, P = 0.043) and late period (days 10–13, right; n = 24 sessions in 6 rats, pre-training: 0.41 ± 0.014 vs. post-training: 0.42 ± 0.015, two-sided paired *t*-test, t_23_ = –1.22, P = 0.23). Mean ± s.e.m. **h**, Comparisons of the mean of top 10% reactivation Rs in SWR^+^ between pre- versus post-training sleep during the early (days 1—4, left; n = 24 sessions in 6 rats, pre-training: 0.73 ± 0.020 vs. post-training: 0.77 ± 0.019, two-sided paired *t*-test, t_23_ = –4.16, P = 4.1 × 10^–4^) and late period (days 10–13, right; n = 24 sessions in 6 rats, pre-training: 0.81 ± 0.016 vs. post-training: 0.82 ± 0.014, two-sided paired *t*-test, t_23_ = –1.48, P = 0.15). Mean ± s.e.m.

We then asked whether SWRs contained reactivations of task-relevant neuronal activity patterns. Using a computational approach of sleep-associated ‘reactivation’ of awake ensemble patterns^18,21,46^, we examined whether SWRs demonstrate increased strength of ensemble reactivations in M1. We used the top three GPFA factors calculated from the population activity during the awake performance (Fig. 3) to identify NREMS reactivations (Methods and Fig. 5a). We used a single fixed window which demonstrated the highest similarity to the awake template to measure reactivation strength (approximately 200 ms after SWR onset; Extended Data Fig. 8a–c and Methods). We then computed a ‘reactivation *R*’ between each reactivation event and the reach template (Fig. 5b; time courses over learning in Extended Data Fig. 8d). When using templates built on spike-shuffled data, we did not find a significant change (Extended Data Fig. 8d). We calculated reactivation *R* values for all SWRs; the mean reactivation *R* value in a single session was chosen to compare SWR^+^ versus SWR^−^ (shown as ΔReactivation *R* in Fig. 5c).

Mean reactivation *R* values in post-training sleep were significantly higher during the SWR^+^ compared with the SWR^−^ condition during early sessions, days 1–4 (*n* = 24 sessions in 6 rats, SWR^+^: 0.38 ± 0.020 versus SWR^−^: 0.34 ± 0.022, two-sided paired *t*-test, *t*_23_ = −3.65, *P* = 1.3 × 10^−3^; Fig. 5d and Extended Data Fig. 8e for the late sessions, days 10–13). We also conducted two analyses to account for potential changes during NREMS SWRs and coupled SOs. First, the mean firing rate for SWR and SO epochs early and late did not change significantly (Extended Data Fig. 7b). The mean firing rate after SWRs (200 ms window) was not different for early versus late sessions (M1, days 1–4, *n* = 24 sessions in 6 rats: 8.03 ± 0.89 Hz versus days 10–13, *n* = 24 sessions in 6 rats: 8.89 ± 1.10 Hz, LME with two-sided *t*-test, *t*_46_ = 1.16, *P* = 0.25; PFC, days 1–4, *n* = 24 sessions in 6 rats: 8.57 ± 1.04 Hz versus days 10–13, *n* = 24 sessions in 6 rats: 7.02 ± 1.09 Hz, LME with two-sided *t*-test, *t*_46_ = −1.49, *P* = 0.14). Second, we also shuffled the spiking data within a single SWR spiking epoch (Fig. 5b and Extended Data Fig. 8d). This preserved the mean firing rate within SWR epochs but altered the temporal structure. Using this approach, we did not find evidence for increased reactivation strength after shuffling. Lastly, the mean firing rate in −0.75 to 0.75 s from SO up-states for early sessions was not different from late sessions (M1, days 1–4, *n* = 24 sessions in 6 rats: 7.78 ± 0.85 Hz versus days 10–13, *n* = 24 sessions in 6 rats: 8.74 ± 1.11 Hz, LME with two-sided *t*-test, *t*_46_ = 1.29, *P* = 0.20; PFC, days 1–4, *n* = 24 sessions in 6 rats: 8.23 ± 1.02 Hz versus days 10–13, *n* = 24 sessions in 6 rats: 6.62 ± 1.05 Hz, LME with two-sided *t*-test, *t*_46_ = −1.57, *P* = 0.12).

We also found that post-training sleep reactivations were greater than those for pre-training sleep for early sessions; this was not the case for late sessions (Extended Data Fig. 8g,h shows two different comparisons between pre-training versus post-training sleep). This is consistent with past studies which show that reactivations are greater in the post-training sleep^19,47^. Moreover, the mean reactivation *R* values in post-training sleep were significantly higher during the SWR^+^SO^+^ compared with the SWR^+^SO^−^ (*n* = 24 sessions in 6 rats, SWR^+^SO^+^: 0.38 ± 0.021 versus SWR^+^SO^−^: 0.35 ± 0.023, two-sided paired *t*-test, *t*_23_ = −2.78, *P* = 0.011; Fig. 5e and Extended Data Fig. 8f for the late sessions). Together, these findings indicate that stronger M1 reactivations of awake task patterns are highly associated with the presence of SWRs in the early training period.

## Re-engaging hippocampal–cortical interactions

**Fig. 6:**
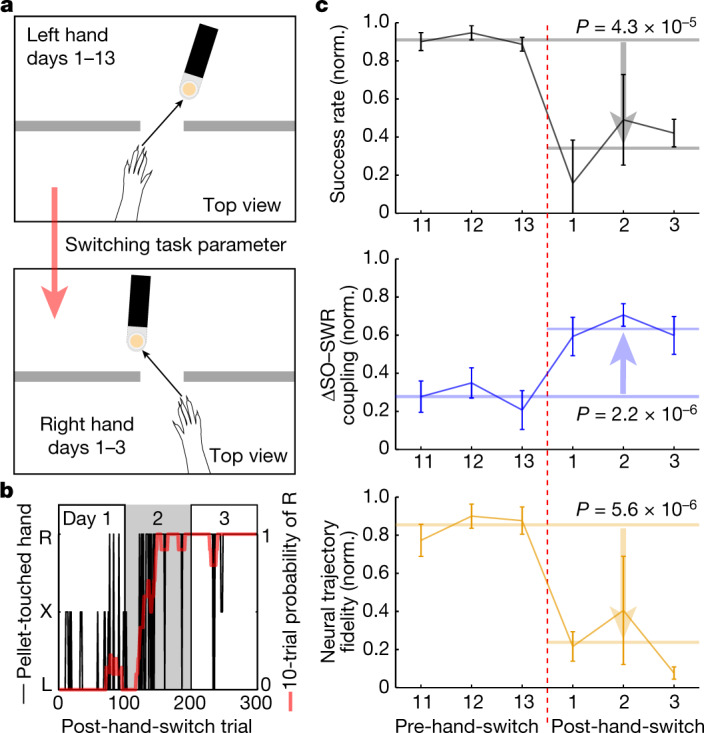
Switching reach-hand increases M1 SO–SWR coupling. **a**, Cartoon of hand-switch experiment. The pellet was located at one side of the wall opening to train animals to use only the dominant hand throughout approximately 13 d. Then, animals were trained to use the opposite hand by locating the pellet on the other side of the wall opening. **b**, Profile of hand use over time (black; L, left; R, right; X, no try) during the post-hand-switch sessions in an example animal. It shows the 10-trial probability of right-hand use (red). **c**, Post-hand-switch changes in pellet retrieval success rate (top), ΔSO–SWR coupling in M1 (middle) and neural trajectory fidelity in M1 (bottom). Horizontal lines indicate the mean during days 11–13 pre-hand-switch (long, *n* = 15 sessions in 5 rats) or during days 1–3 post-hand-switch (short, *n* = 12 sessions in 5 rats); there was a significant increase or decrease after hand-switch (comparison of the session means using LME with two-sided *t*-test, success rate: *t*_25_ = −4.81, *P* = 4.3 × 10^−5^, ΔSO–SWR coupling: *t*_25_ = 6.11, *P* = 2.2 × 10^−6^, neural trajectory fidelity: *t*_25_ = −5.74, *P* = 5.6 × 10^−6^). These metrics after hand-switch were normalized relative to the maximum-to-minimum before the hand-switch; this allows us to directly compare the changes after hand-switch compared with the above results. Mean ± s.e.m.

Systems consolidation is usually presented as a transfer to cortex with subsequent hippocampal independence. We wondered whether there was a possibility of continuous dialogue. We thus investigated how alterations in task parameters that require new exploration (in this case, need to switch hands) change hippocampal–cortical dialogue. After long-term training using the left hand, in five of the six rats, we changed the pellet location such that the right hand was now required (Fig. 6a). The box and the contextual cues that predicted door opening and task start remained constant. As these animals were overtrained, early exploration was associated with left hand movements; they did not switch to the right hand until session end (Fig. 6b).

As expected, the success rate significantly decreased (pre-switch, *n* = 15 sessions in 5 rats: 0.91 ± 0.023 versus post-switch, *n* = 12 sessions in 5 rats: 0.34 ± 0.13, LME with two-sided *t*-test, *t*_25_ = −4.94, *P* = 4.3 × 10^−5^; normalized to the pre-switch maximum and minimum in Fig. 6c). Notably, in the following NREMS, ΔSO–SWR coupling in M1 was elevated (pre-switch, *n* = 15 sessions in 5 rats: 0.27 ± 0.050 versus post-switch, *n* = 12 sessions in 5 rats: 0.64 ± 0.052, LME with two-sided *t*-test, *t*_25_ = 6.11, *P* = 2.2 × 10^−6^). We also found that this elevated ΔSO–SWR coupling was associated with increased variability in GPFA neural trajectories in M1, that is, lower fidelity (pre-switch, *n* = 15 sessions in 5 rats: 0.85 ± 0.042 versus post-switch, *n* = 12 sessions in 5 rats: 0.27 ± 0.12, LME with two-sided *t*-test, *t*_25_ = −5.74, *P* = 5.6 × 10^−6^). Thus, our results suggest that, even though the spatial cues in the box and contextual task cues were constant, motor exploration, even after systems consolidation, appeared to alter hippocampal–cortical dialogue during NREMS.

## Discussion

Our results using skill learning provide insights into the precise spatiotemporal coordination of multiple areas throughout systems consolidation. Our observed task-specific reactivations during early manifold exploration suggest a close link between phase-locking of cortical SOs, hippocampal SWRs and cortical reactivations. These results also suggest that hippocampal SWRs may represent a preserved mechanism of consolidation for both declarative and procedural memories.

Although skill learning was initially considered to occur independently of the hippocampus^24,35^, recent work indicates that the hippocampus may be recruited early^25,26^. Our results further show how temporally precise feedback between hippocampus and cortex might enable two-stage consolidation and stabilization of M1 neural trajectories. We wondered how we could reconcile this with classic studies of patients with medial temporal lobe lesions who demonstrate skill learning despite lack of explicit recall^48^. Although such patients could clearly learn new skills, their performance levels were somewhat worse than age-matched controls^49^. Thus, although medial temporal lobe-dependent processing may not be completely necessary and remaining systems can partially compensate, it seems increasingly possible that the hippocampus can facilitate consolidation of skills.

We investigated how the hippocampus and PFC might support motor learning. In most experimental skill learning tasks (and in real-world settings), there are contextual cues that inform timing and action selection. In our reaching task, animals must learn to associate allocentric spatial aspects of the box (for example, door and pellet location) with a reaching action. There is also a sound to predict door opening. It is possible that PFC activity patterns^50^ support M1 in predicting task rules and the timing of reach. When considering these components of ‘motor learning’, there may also be overlap with instrumental learning (that is, association of actions and outcomes), which may involve hippocampal processing^51^. Substantial future work is required to uncover the precise causal roles of these regions in skill learning and adaptation.

We found that changes in the variability of task activity were temporally associated with stepwise changes in cross-area coupling. Feedback based on task performance and manifold variability may be a key driver to cease hippocampal learning-related engagement and shifts in coordination. Our results also indicate that dialogue across these systems does not end after systems consolidation. In previous studies, reset of pre-existing connectivity stabilized by consolidation was found to facilitate new learning^52,53^. It is quite possible that ‘unlearning’ and entering an exploratory state is supported by sleep processing.

The original notion of systems consolidation did not specify precisely what integration into cortex entailed. After apparent hippocampal disengagement, we observed steady increases in fidelity. It is possible that our observed stages are related to fast and slow learning^54,55^. Steady increases in fidelity could be due to greater engagement of the striatum^56^, particularly during NREMS^33^. Moreover, although the mechanisms driving shifts from stage I to II are unclear, reward-based processing and dopamine dynamics may be important^57^. Interestingly, SWR-associated reactivations are also found in areas linked to reward processing^58^. How consolidation interacts with reward systems is an intriguing line of inquiry.

## Methods

### Animals/surgery

Experiments were approved by the Institutional Animal Care and Use Committee at the San Francisco VA Medical Center. We used a total of six adult Long-Evans male rats (300–400 g; Charles River Laboratories). No statistical methods were used to predetermine sample sizes, but our sample sizes are similar to those reported in previous publications^18–20^. Animals were kept under controlled temperature and a cycle with 12 h light and 12 h dark (lights on at 6:00). Surgeries were performed under isoflurane (1–3%) anaesthesia and body temperature was maintained at 37 °C with a heating pad. Atropine sulfate was also administered intraperitoneally before anaesthesia (0.02 mg per kg of body weight). We implanted 64-channel multishank microwire arrays for recording LFP/spike activity (Fig. 1b). One strand of 32-channel arrays was lowered down to 1.4–1.8 mm in layer 5 of the M1 (histology confirmation in Extended Data Fig. 1a) in the forelimb area centred at 3.0 mm lateral and 0.5 mm anterior from the bregma. The other strand of 32-channel arrays was lowered to 3.5–4.0 mm of PFC centred at 1.0 mm lateral and approximately 3.5–4.0 mm anterior from the bregma. In addition, we implanted 32-channel single-shank linear electrode arrays for recording LFP in the CA1 area of dorsal hippocampus; the tip of electrode arrays was lowered with the angle of 20 degrees to target the coordination of −3.6 mm in AP, 3.0 mm in ML and 4.0 mm in DV from the bregma. Then we adjusted the tip position approximately 200 μm to ensure the best spike recordings in potential CA1 areas (specific three channels ranging 250 μm longitudinally). The reference and ground wires were wrapped around a screw inserted in the midline over the cerebellum. The final localization of depth was based on the quality of recordings across the array at the time of implantation. The post-operative recovery regimen included administration of buprenorphine at 0.02 mg kg^−1^ and meloxicam at 0.2 mg kg^−1^. Dexamethasone at 0.5 mg kg^−1^ and trimethoprim sulfadiazine at 15 mg kg^−1^ were also administered postoperatively for 5 d. All animals were allowed to recover for at least 7 d with the same regimen as described above before the start of experiments. The food restriction was conducted from 2 d before the start of the experiment until the completion of the experiment; half of the standard amount of food (50 g kg^−1^) was given to an animal during this period. During behavioural assessments, we monitored the animals and ensured that their body weights did not drop below 90% of initial weight. Data collection and analysis were not performed blind to the conditions of the experiments.

### Electrophysiology

We conducted a.c.-coupled recordings and recorded extracellular neural activity using double-stranded 64-channel microwire electrode arrays (33 μm diameter, 250 μm spacing, 2.5 mm length and 4 rows in a single 32-channel strand; 33 μm diameter, 250 μm spacing, 5.0 mm length and 4 rows in the other single 32-channel strand; standard polyimide-coated tungsten microwire arrays from Tucker-Davis Technologies (TDT)) and 32-channel linear electrode arrays (15 μm thickness, 10 mm length, 50 μm spacing and single shank; standard silicone probes from NeuroNexus). All electrode arrays showed similar quality of LFP (for example, LFP amplitude and noise level). We recorded spike and LFP activity using a 128-channel RZ2 bioamp processor (TDT) and 128-channel neurodigitizer (digital PZ5).

Spike data were sampled at 24,414 Hz and LFP data at 1,018 Hz. ZIF-clip-based headstages (TDT) for 64/32-channel electrode arrays with a unity gain and high impedance (approximately 1 G) were used. Only clearly identifiable units with good waveforms and a high signal-to-noise ratio were used. The remaining neural data (for example, filtered LFP) were recorded for offline analyses at 1,018 Hz. Behaviour-related timestamps (that is, trial onset and trial completion) were sent to the RZ2 analog input channel using a digital board and then used to synchronize to neural data in the offline analyses. The LFP was analysed after removing obvious artefacts (>10 s.d.) and excluding bad channels. We used all M1 channels excluding bad channels covering the forelimb-related area (M1 channel count: 23–32 channels, 29.7 ± 2.2, mean ± s.d.). Although the coverage of 32-channel microwires in PFC was large, we did not make any selection of particular channels to include specific PFC areas (for example, infralimbic cortical area known to be important for rule learning), because we focused on more the global activity (that is, SOs) in PFC rather than spike ensemble activity. Thus, we also used all good channels in PFC (PFC channel count: 18–32 channels, 28.3 ± 3.2, mean ± s.d.). However, we located PFC channels to collect spikes and LFP in the medial PFC including the infralimbic area.

### Behaviour

After recovery from probe implantations, animals were typically handled for several days (3–5 d) before the start of experimental sessions, that is, ‘day 1’ of long-term recording in which rats showed a successful trial in motor reach-to-grasp task. Animals were acclimated to a custom plexiglass behavioural box during this period without motor training. The box was equipped with a big door at one end for the animal entry and a tiny door at the side for the motor training. After the initial acclimation period, animals were trained for a reach-to-grasp single-pellet task. Typically, animals showed a successful trial at days 1–3 after the start of motor training; the sessions before the day of the first successful trial were not considered as long-term learning sessions shown as days 1–13 in Fig. 1a. The first reach training session (day 1 of long-term motor learning in Fig. 1a) was during days 11–13 (12.5 ± 0.84, mean ± s.d.) after probe implantations; probe implantations were performed contralateral to the preferred hand. Animals were trained to a plateau level of performance in a reach-to-grasp single-pellet task during 12–13 d (12 d in one animal and 13 d for the other five animals). In a single session of the motor task, pre-training sleep, reach training and post-training sleep were monitored in sequence. The reach-to-grasp task has been used as a sensitive measure of motor function; it requires reaching, grasping and retrieving a single pellet located at a distance outside of the behavioural box^13,14,59^. The pellet was located at one side of the wall opening to train animals to use only the dominant hand (Fig. 6a).

Across animals, we compared relative pellet retrieval success rate in the reach-to-grasp task, using normalized metrics relative to the maximum and minimum success rate; that is, the normalized metric ranged between one and zero corresponding to the maximum and the minimum of the absolute metric, respectively. However, in the hand-switch experiment (Fig. 6), the reach performance after the hand-switch was normalized relative to the maximum-to-minimum before the hand-switch; this was to directly compare the changes after hand-switch with the levels before hand-switch. We monitored electrophysiology, that is, LFP/spike only during the pre-training sleep, reach training and post-training sleep, not during 24 h of a single day. This typically totalled a period of 4–5 h per day starting at 8:30 to 9:30 in the behavioural box. After completing motor tasks and sleep sessions in the behavioural box, animals were placed in the home cage without electrophysiology monitoring.

For the behavioural task, we used an automated reach-box, controlled by custom MATLAB scripts and an Arduino microcontroller. This setup for the reach-to-grasp task required minimal user intervention, as described previously^60^. Each trial consisted of a pellet dispensed on the pellet tray, followed by a beep indicating that the trial was beginning; this was followed by the door opening. Animals then had to reach their arm out, grasp and retrieve the pellet. A real-time ‘pellet detector’ using an infrared detector centred over the pellet was used to determine when the pellet was moved, which indicated that the trial was over and the door was closed. All trials were captured by video, which was synced with the electrophysiology data using the Arduino digital output. The video frame rate was 75 Hz. The reach performance (the number of accurate pellet retrievals/the total number of trials × 100%) was determined manually from a recorded video. The reach performance was used as a measure of motor learning and memory consolidation across sessions. We also examined task errors, that is, the change in success rate (Extended Data Fig. 2e and Fig. 1f). This was calculated by subtracting the 2 d history success rate from the current day success rate; the 2 d before day 1 was set to zero. For example, the mean success rate of day 5 was subtracted by the mean success rate of days 3–4. After computing the task errors using the absolute success rate, this was normalized in the same manner as for the absolute success rate.

### Identification of NREMS oscillations

LFP activity was recorded using 64-channel microwire electrode arrays and 32-channel linear electrodes arrays (see above). The LFP was analysed after removing obvious artefacts (>10 s.d.) and excluding bad channels. Identification of NREMS epochs was performed by classification based on power spectral density of the LFP. This study focused on the NREMS epochs detected using M1 LFP. However, we further conducted NREMS detections using all three areas (that is, PFC, M1 and hippocampal CA1). The total duration of NREMS was not significantly different across the recorded areas (Extended Data Fig. 1c). Moreover, cutting out the last NREMS epoch did not affect the transition trend in ΔSO–SWR coupling (Extended Data Fig. 1d). In detail, regarding NREMS epoch detection, the LFP trace was segmented into non-overlapping 6 s epochs. In each epoch, the power spectral density was computed and averaged over the slow-wave frequency band (0.1–4 Hz, also called the delta band) and gamma frequency bands (30–60 Hz). Then a *k*-means classifier was used to classify epochs into two clusters, NREMS and rapid eye movement sleep (REMS)/awake; REMS and awake were not classified and NREMS was focused on in this study. Sleep epochs less than 30 s were excluded from NREMS epochs. The identified NREMS durations were not different between the early period and late period of motor learning. The identified NREMS epochs were verified by visual assessment of the LFP activity. During the NREMS period with high delta power (0.1–4 Hz), strong down- and up-states dominated. Thus, we assessed whether our detected NREMS epochs contained a high-amplitude and slow LFP fluctuation distinguished from a low-amplitude and high-frequency LFP during the awake period. Moreover, we visually assessed whether there were substantially many wrong detections of NREMS epoch. In other words, we assessed if a high-amplitude and slow-wave LFP epoch was not included in the detected NREMS epochs. These power-based sleep detections showed a close match to the video-based detections^18^; the number of pixels that changed intensity frame to frame in each pair of consecutive frames was computed from a recorded video (1 Hz frame rate using Microsoft LifeCam Cinema Webcam) during the sleep block; these values were then integrated over an epoch of 40 s. If the integrated value was higher than a threshold, that epoch was identified as sleep; the threshold was chosen by comparing detection results and visual assessment of the recorded video.

In offline analysis, SOs and spindles in the deep layer of PFC and M1 were detected with the algorithm used in previous studies^18,20,33,61^. The recorded layer of PFC and M1 was confirmed with histology (Extended Data Fig. 1a). We also confirmed that the waveform and spike activity during the detected SOs were comparable to the previous publications^18,20,38,62^. The LFP average across all recording channels excluding bad channels was filtered in the delta band (0.1–4 Hz) through two independent filterings: the high-pass Butterworth filter (second order, zero phase-shifted, with a cutoff at 0.1 Hz) was applied and then followed by the low-pass Butterworth filter (fifth order, zero phase-shifted, with a cutoff at 4 Hz). The individual orders of the high-pass and low-pass filters were estimated through a conventional minimum-order design; it required to meet maximum passband ripple of 3 dB and minimum stopband (presumed 0.02 Hz for high-pass filter and 6 Hz for low-pass filter) attenuation of 15 dB. Next, all positive-to-negative zero crossings during NREMS were identified, along with the previous peaks, the following troughs and the surrounding negative-to-positive zero crossings. Then the positive threshold (the top 15 percentile of the peaks) and the negative threshold (the bottom 40 percentile of the troughs) were, respectively, defined for the down-states (that is, silence of neural spiking) and up-states (that is, increased spiking); in Fig. 1b, horizontal dashed lines indicate the thresholds detecting SO up-/down-states. Each identified wave was considered an SO if the trough was lower than the negative threshold (that is, up-state), the peak preceding that up-state was higher than the positive threshold (that is, down-state) and the duration between the peak and the trough was between 150 ms and 500 ms (Fig. 1b).

For spindles detection in PFC and M1, the LFP was first *Z-*scored in each channel and averaged across all good channels as for the SOs detections. The LFP average was filtered in a spindle band (10–15 Hz) through two independent zero phase-shifted filterings (Extended Data Fig. 5a): the high-pass Butterworth filter (sixth order, zero phase-shifted, with a cutoff at 10 Hz) was applied and then followed by the low-pass Butterworth filter (eighth order, zero phase-shifted, with a cutoff at 15 Hz). The individual orders of the high-pass and low-pass filters were estimated through a conventional minimum-order design; it required to meet maximum passband ripple of 3 dB, and minimum stopband (presumed 7 Hz for high-pass filter and 19 Hz for low-pass filter) attenuation of 15 dB. We computed a smoothed envelope of this signal, the magnitude of the Hilbert transforms with convolving by a Gaussian window (200 ms). Next, we determined two thresholds for spindle detection based on the mean (*μ*) and standard deviation (*σ*) of the spindle band LFP during NREMS; the upper and lower thresholds were set *μ* + 2.5 × *σ* and *μ* + 1.5 × *σ*, respectively; in Extended Data Fig. 5a, horizontal dashed lines indicate the thresholds detecting spindle peak and spindle period, respectively. Epochs in which the spindle power exceeded the upper threshold for at least one sample and in which the spindle power exceeded the lower threshold for at least 500 ms were considered spindles. Each epoch for which the spindle power exceeded the lower threshold was considered the start and stop of the spindle; the duration of each spindle was based on these values as well.

For SWRs detection in hippocampus, the LFP was first *Z*-scored in each channel and averaged across three channels located on the dorsal CA1 area; during the surgery the electrode tip position was adjusted approximately 200 μm to ensure the best spike recordings in potential CA1 areas (specific three channels ranging 250 μm longitudinally). The LFP average was filtered in the ripple band (150–250 Hz) through two independent zero phase-shifted filterings (Fig. 1b); the high-pass Butterworth filter (eighth order, zero phase-shifted, with a cutoff at 150 Hz) was applied and then followed by the low-pass Butterworth filter (tenth order, zero phase-shifted, with a cutoff at 250 Hz). We computed a smoothed envelope of this signal, the magnitude of the Hilbert transforms with convolving by a Gaussian window (20 ms). Next, we determined two thresholds for SWRs detection based on the mean (*μ*) and standard deviation (*σ*) of the SWR band LFP during NREMS; the upper and lower thresholds were set *μ* + 4 × *σ* and *μ* + 1 × *σ*, respectively; in Fig. 1b, the horizontal dashed line indicates the lower threshold. Epochs in which the SWR power exceeded the upper threshold for at least one sample and in which the SWR power exceeded the lower threshold for at least 50 ms were considered SWRs. Each epoch for which the SWR power exceeded the lower threshold was considered the onset and end of the SWR; the duration of each SWR was based on these values as well.

### Sleep oscillations coupling analyses

We analysed the temporal coupling of SWRs relative to SOs. For the coupling of SWRs to SOs (SO–SWR coupling; Fig. 2a), each SWR was linked to the closest SO. The time difference between the peak of the SWR and the up-state of the linked SO was measured for each detected SWR (Δ*T*_SO–SWR_). If Δ*T*_SO–SWR_ was between −0.75 s and 0.75 s (that is, nesting time window, black box in Fig. 2b and Extended Data Fig. 3a), that SWR event was considered an SO-coupled SWR. To quantitatively assess the changes in the temporal coupling of SWRs to SOs, we specifically measured the following: the time lag of the SWR from the closest SO (Δ*T*_SO–SWR_) was measured for each SWR event and the rate of SWRs for which Δ*T*_SO–SWR_ was within the nesting time window was measured; that is, the number of SO-coupled SWRs/the total number of SWRs × 100% (Fig. 2b,c). To quantify ‘training-related’ change in SO–SWR coupling over days, we measured ‘ΔSO–SWR coupling’ as the difference between post-training sleep and pre-training sleep (Fig. 2d). We also measured the temporal coupling of SOs observed in PFC and M1, focusing on post-training because the temporal coupling of SOs changes with a slower timescale compared with SO–SWR coupling. For the coupling of PFC SOs and M1 SOs (PFC–M1 SO coupling; Fig. 1c), each M1 SO was linked to the closest SO. The time difference between the up-state of M1 SO and the up-state of the linked PFC SO was measured for each detected M1 SO (Δ*T*_SO_). If Δ*T*_SO_ was between −0.2 s and 0.2 s, that M1 SO was considered a coupled SO. Otherwise, M1 SO was considered an uncoupled SO. We then calculated PFC–M1 SO coupling as the number of coupled SOs in PFC and M1 divided by the total number of SOs (coupled + uncoupled) in M1 (Fig. 1c).

The SWR-shuffled condition in Extended Data Fig. 3c represents temporal coupling that was measured using the intact SOs and temporally disrupted structure of SWRs. The temporal profile of SWRs in a sleep session was disrupted by circular permutation. The measurement of SO–SWR coupling with circular permutation was repeated 1,000 times in each session and the mean of those 1,000 measures was reported. The same shuffling process was applied to the PFC SO-shuffled M1 SO coupling in Extended Data Fig. 2b. In this case, temporal coupling was measured using the intact PFC SOs and temporally disrupted structure of M1 SOs.

The effect of SWRs on the temporal coupling between PFC SO and M1 SO was examined using the same measurement of Δ*T*_SO_ in Fig. 4a as well as on the motor memory reactivations in Fig. 5d. We examined it during the SWRs (SWR^+^) and the random events (SWR^−^). The SWR^+^ condition represents 1 s time epochs following an SWR onset. The SWR^−^ was defined as the 1 s time epoch following an event that was randomly selected during NREMS; the total number of events of the SWR^−^ condition was set to the number of SWRs for the SWR^+^ condition in each same session. We also examined the intercortical communications and the reactivations during the period of SWRs (Figs. 4d and 5e, respectively); specifically, we compared the SWRs with an SO within 1 s (SWR^+^SO^+^) versus the SWRs with no following SO in 1 s (SWR^+^SO^−^). For this category, we focused on more M1 SOs rather than PFC SOs. Thus, the SWR^+^SO^+^ was defined as an SWR followed by an M1 SO within a 1 s period after that SWR onset, whereas the other SWRs without a following M1 SO within 1 s were considered as SWR^+^SO^−^.

We also analysed the temporal coupling of spindles relative to SOs. For the nesting of spindles to SOs (SO–Spindle coupling; Extended Data Fig. 5b), each spindle was linked to the closest SO. The time difference between the peak of the spindle and the up-state of the linked SO was measured for each detected spindle (Δ*T*_SO–Spindle_). If Δ*T*_SO–Spindle_ was between −0.5 s and 1.0 s (that is, nesting time window), that spindle event was considered an SO-nested spindle. To quantitatively assess the changes in the temporal coupling of spindles to SOs, we specifically measured as the following: time lag of spindle from the closest SO (Δ*T*_SO–Spindle_) was measured for each spindle event and the rate of spindles for which Δ*T*_SO–Spindle_ was within the nesting time window was measured; that is, the number of SO-nested spindles/the total number of spindles × 100% (Extended Data Fig. 5c,d). We observed ‘triple-coupling’ (that is, precise coupling of SWR, SO and spindle; Extended Data Fig. 4b): the percentage of SWRs locking with both SO and spindle between −1 s and 1 s from an SWR peak. We also observed ‘quadruple-coupling’ (that is, precise coupling of SWR, coupled SOs in M1 and PFC, and spindle; Extended Data Fig. 6e): the percentage of SWRs locking with PFC–M1-coupled SOs, and spindle between −1 s and 1 s from an SWR peak.

### Spike activity during events

We initially used an online sorting programme (SpikePac, TDT). We then conducted offline sorting using MountainSort for six rats^63^. Briefly, MountainSort is a spike-sorting software that uses an automatic algorithm, which compares clusters of data and identifies single units using one-dimensional projections. After the automatic sorting using MountainSort, a minimal manual merging and rejection of clusters were performed. Only clearly identifiable units along with sessions of a single day, with good waveforms and a high signal-to-noise ratio, were used. There was no significant change in the number of recorded units across long-term motor learning in both M1 and PFC (the number of units per session in M1, early, *n* = 24 sessions in 6 rats: 30.54 ± 1.35 per session versus late, *n* = 24 sessions in 6 rats: 29.00 ± 0.97 per session, LME with two-sided *t*-test, *t*_46_ = −1.22, *P* = 0.23; the number of units per session in PFC, early, *n* = 24 sessions in 6 rats: 25.50 ± 1.61 per session versus late, *n* = 24 sessions in 6 rats: 25.46 ± 1.68 per session, LME with two-sided *t*-test, *t*_46_ = −0.026, *P* = 0.98).

To assess spike activity modulation during awake training epochs and sleep oscillations, we analysed PETH. After spikes were time-locked to event reference times (for example, reach onsets during task or SWR onsets during sleep), the PETH (bin length = 15 ms) was estimated. The PETH during a reach onset was calculated in an approximately 600–1,200 ms window depending on the mean reach-to-pellet-touch duration for each animal (−200 to 400 ms for 4 rats, −200 to 600 ms for 1 rat and −200 to 1,000 ms for 1 rat from a reach onset; see also the ‘GPFA’ section below). The PETH during an SWR onset was calculated in 195 ms and 1,000 ms windows after the SWR onset, respectively, for the reactivation and the CCA analysis (see the detailed method of window size selection in the ‘Memory reactivation analyses’, and more details in the ‘CCA’ section below). The PETHs in Fig. 3 and Fig. 5 are shown after normalization in each unit by subtraction of mean and then division by standard deviation in each event window.

The spike-shuffled condition in Extended Data Fig. 6c and Extended Data Fig. 8d was conducted using the circular permutation in an individual unit. Among the recorded unit population, the timing of spike events in the first unit was permutated around an event time in a circular way and then it was repeated along with the recorded units as well as along with the monitored events. The time window for the circular permutation was approximately three times longer than the analysis window described above for the GPFA and the reactivation analysis; circular permutation windows were −600 to 1,000 ms for the GPFA analysis and −200 to 400 ms for the reactivation analysis. The one set of circular permutations in each session was repeated 1,000 times and the mean of those 1,000 measures was reported.

### CCA

To identify a shared cross-area subspace (that is, communication subspace between PFC and M1 in Fig. 4), we used CCA which identifies maximally correlated linear combinations between two groups of variables^40^. As illustrated in the cartoon in Fig. 4b, CCA allows us to identify cross-area activity axes (CV) on which projections of local PFC neural dynamics (red) and M1 neural dynamics (black) are maximally correlated during SWRs. CCA was carried out using the MATLAB function ‘canoncorr’. Neural data in PFC and M1 during SWRs were binned at 15 ms, and data during 1 s from SWR onsets were concatenated across events and mean subtracted. Then CCA models were fit to these data in each session. CCA produces as many CVs as the number of neurons in the smaller population (for example, if there are 10 PFC neurons and 15 M1 neurons, then CCA will fit 10 CVs). For evaluating cross-area activities, only the top CV explaining maximally correlated cross-area activity among CVs was used, as this provided a consistent dimensionality across datasets, and a signal with both magnitude and sign. Then we calculated ‘cross-area correlation (*R*)’: the correlation coefficient between PFC cross-area activity and M1 cross-area activity was computed for SWR periods in Extended Data Fig. 7c as well as for the periods of SWR^+^SO^+^ and SWR^+^SO^−^ in Fig. 4c. The stronger communication is demonstrated as alignments of cross-area activities with ‘communication subspace’ (diagonal dashed line in Fig. 4c) resulting in a higher cross-area *R* value. On the contrary, the weaker communication or independent activity either in PFC or M1 is demonstrated as alignments with the PFC subspace (vertical dashed line) or M1 subspace (horizontal dashed line) resulting in a lower cross-area *R* value.

### GPFA

To characterize single-trial representations of population spiking activity in Fig. 3, we used GPFA^29^ to find low-dimensional neural trajectories for each trial. GPFA analyses were carried out for an individual area of PFC or M1 using the MATLAB-based DataHigh (v.1.3)^64^. As shown using the diagram in Fig. 3a, this method allows us to decompose neural population activity (*x* = *μ* + *x*^shared^ + *x*^private^; in which *μ* represents mean spike firing rate computed in each neuron) into shared activity (*x*^shared^ = *Uz*; in which *U* and *z* represent shared factor weight and shared factor activity, respectively) and private activity (*x*^private^); the top cartoon in Fig. 3a is shown using two neurons (N_1_ and N_2_). The bottom cartoon illustrates a transition from ‘manifold exploration’ in early learning periods (for example, days 3 and 4) characterized with highly variable shared activity to ‘manifold consolidation or stabilization’ in the late learning period (for example, days 9 and 13) characterized with the emergence of a manifold and stable neural trajectories over long days.

GPFA was performed with a time bin of 15 ms and the optimal dimensionality of 6 which was determined by the method of ‘leave-neuron-out strategy’ proposed in the previous study^29^; the first factor accounts for the largest variance in the neural population activity during task training. For each session, we concatenated binned spike trains for each neuron across trials. This concatenated spike train was *Z*-transformed to account for neurons with high firing rates. Then the *Z*-transformed spike trains were placed into a two-dimensional matrix organized by neurons (*x*) and time (*y*, the number of time bins). From this spike count matrix, the shared factor weight (also called GFPA factors) and shared factor activity (projections of the *Z*-transformed spike trains onto the GPFA factors) were computed. The top three factors were used for the analyses; they accounted for >85% of shared variance explained in respective M1 and PFC in each session (example trajectories in M1 in an animal, Extended Data Fig. 6a,b). We confirmed that shared-over-total variance, that is, shared variance divided by the sum of shared and private variance, showed a robust increase during learning (Extended Data Fig. 6c). However, the shared-over-total variance with no significant change in the spike-shuffled condition (see above for the details about the circular permutation) supports the idea that the temporal pattern of neural ensemble resulted in the robust learning in neural dynamics and not the simple change in population firing rate. We recalculated the low-dimensional manifold (that is, GPFA factors) every session because recorded units were not able to be held across days. To compensate for possible variations of low-dimensional manifolds, the estimated manifolds were aligned to the average manifold of the final 3 d by using Procrustes alignment (MATLAB function ‘procrustes’)^41,65^.

We examined the consistency and variance of neural trajectories over long-term task learning. First, in an individual animal, we calculated ‘GPFA correlation’: the correlation coefficient of a single-trial neural trajectory with the optimal trajectory calculated by averaging the neural trajectories of the three sessions in which the animal showed the best performance over the 13 d task training. It provided a robust measure of neural optimality for a successful task as the mean and standard deviation of this measure increased and decreased, respectively, during the long-term learning (Extended Data Fig. 6d). To quantify the optimality as well as stabilization of neural trajectories, we then measured ‘neural trajectory fidelity’, that is, signal-to-noise ratio^42–44^, of GPFA correlations across trials. This measure was calculated by the mean over the standard deviation of GPFA correlations across trials and compared the level of a desired neural trajectory (‘signal’) with the level of deviation from the optimal trajectory (‘background noise’); it measures both strength and consistency of GPFA correlations across all trials including both successful and failed trials. For this measurement, ‘high fidelity’ represents low representational variability and stable neural trajectories, and ‘low fidelity’ represents greater representational variability. We then define low fidelity as ‘manifold exploration’, because there is large variation in neural trajectories. With practice and changes in sleep coordination, we see a transition to steady increases in fidelity. We define this as ‘manifold consolidation’.

GPFA was performed specifically for the period near the reach-to-grasp: depending on the mean duration from reach onset to pellet touch in each animal, for M1 population activity it was between −200 ms and 400 ms from reach onset for four animals, between −200 and 600 ms from reach onset for one animal, and between −200 and 1,000 ms from reach onset for one animal. The same computations were conducted for PFC population activity but using the expanded periods, which were expanded by 100 ms both at the beginning and the end of each epoch for M1. Specifically, for the GPFA correlations, we used neural trajectories during the mean reach-to-grasp period of that session. As this duration varied across trials, sessions and animals, we interpolated each trial such that every trial was the same length (100 values) and then calculated correlation coefficients.

### Memory reactivation analyses

To characterize ensemble reactivations during sleep, we performed an analysis that compared neural activity patterns in M1 during post-training sleep using a template that was created based on neural activity during reach task training. The previous studies typically used principal component analysis to convert a set of observations of neural activity during the task into a set of values of linearly uncorrelated variables called principal components^18–21,66^. In this study we used GPFA factors instead of principal components to directly compare the findings with the concept of neural trajectory in Fig. 3; the basic approach is analogous to the previous studies (Fig. 5a). In detail, we used the top three GPFA factors computed using the spike trains during task trials (see above for the details of GPFA computation using awake activities), that is, the manifold or the template space in which a template of ensemble activity evolves during reach training. The binned spike trains (bin length = 15 ms) during a particular event of post-training sleep (for example, SWR and random event) were then projected onto the ‘template space’, that is, GPFA top three factors defined by the neural activity during reach training; this projection was a linear combination of *Z*-scored binned spike trains from post-training sleep with the template space calculated from the neural activity during reach training. This linear combination has been termed the ‘neural trajectory reactivation’.

As motor memory reactivation is known to be temporally compressed/extended to different degrees relative to an awake pattern^46,67^, we looked for reactivations with different window sizes (75–405 ms corresponding to approximately 0.2–2× compression/extension) as well as with various time lags (0–405 ms) from an SWR onset; we explored the combinations of sizes and time lags to find the best reactivation for each SWR onset (Extended Data Fig. 8a). Here time lag was limited so that the end of prediction windows was not out of the 405 ms period from an SWR onset. In each SWR onset of post-training sleep, the combination showing the maximal correlation with the reach template (mean neural trajectory across the conducted task trials) of that session was selected for the final neural trajectory reactivation for that event; the distributions of window sizes and time lags of the final neural trajectory reactivations are shown in Extended Data Fig. 8b,c. As a simple comparison across trials and sessions, we then used the fixed window size to measure neural trajectory reactivations; the most frequent size and time lag were used for all sessions and trials, that is, 195 ms size and zero time lag from the SWRs onsets. The correlation coefficient between a neural trajectory reactivation during this window and the reach template was then termed the ‘reactivation correlation (*R*)’ of that particular neural trajectory reactivation. We confirmed the robust increase over long-term motor learning (Extended Data Fig. 8d). However, the reactivation *R* did not demonstrate significant changes in the spike-shuffled condition. The goal of the shuffling procedure, done per event epoch, was to maintain the mean firing while disrupting the temporal structure across neurons. This supports the idea that the temporal pattern of the neural ensemble resulted in a robust increase in reactivation *R*.

We examined reactivations for all SWRs; the mean/median of reactivations in a single session was chosen to use. We confirmed that the reactivation *R* values during sleep in observed spike activities of a particular condition were significantly greater than in the spike-shuffled condition, that is, the projections of temporally shuffled neural activity for each unit in each event window onto the template space (GPFA top three factors). The mean/median of the reactivation *R* values computed in each session was then used for the comparison between the SWR^+^ versus the SWR^−^ as well as the SWR^+^SO^+^ versus the SWR^+^SO^−^ condition.

### Across-session analyses over long-term learning

Cross-areas coordination and neural population dynamics were analysed during the long-term motor skill learning in Figs. 1d,f, 2c–e and 3d. To examine the transitions across sessions, we used tertiles; either trials during reach training or events during sleep were divided into tertiles per session. We then convolved the 13 d data (39 tertiles in five animals; 12 d with 36 tertiles in one animal) using a Gaussian window of 3 d (9 tertiles); the data were padded by an average during the first 2 d at the start and by an average during the last 2 d at the end before convolution. As mentioned above, in a single animal, the 12 d motor training was performed; thus, in this animal, days 9–12 were analysed for the late period marked ‘days 10–13’.

To extend the reliability of two-stage prediction beyond the variability in absolute values across multiple animals, the metric was normalized in each animal; the normalized metric ranged between one and zero corresponding to the maximum and the minimum of the absolute metric, respectively. The convolved and normalized data were then fitted to the Sigmoid function having a characteristic ‘S’-shaped curve which allows us to predict two-stage transitions over time with high versus low values. We used a general form of the sigmoid function, in which *U* and *L* were the upper and lower boundaries, respectively, *x*_mid_ was the midpoint parameter (symmetric point of S-shaped curve) and *k* was the slope parameter. The midpoint parameter of the sigmoid function informs the temporal distinction of two stages. The sigmoid function fittings were conducted focusing on days 3–11 for M1 and days 3–13 for PFC because of the delayed transition of cross-area coupling in PFC compared with M1. This information was used to determine the sharpest changes in ΔSO–SWR coupling and PFC–M1 SO coupling and to compare the sequential interactions from the stepwise increase in PFC–M1 SO coupling to the drop in ΔSO–SWR coupling (Fig. 2e). For the success rate in Extended Data Fig. 2f, the convolved and normalized data in the same way for PFC–M1 SO coupling were fitted to the single-exponential function to estimate the decay over days (*P* = 7.5 × 10^−14^).

We also used the piecewise linear regression method to predict two/three linear trends of changes in the PFC–M1 SO coupling (Fig. 1d,e), the changes in success rate (Fig. 1f) and the neural trajectory fidelity (Fig. 3d). The two/three trends were first classified based on the time course of the activity in six rats. Linear slope across points in a moving window was measured using conventional linear regression (MATLAB function ‘regress’). For example, in the neural trajectory fidelity in Fig. 3d, linear slopes were measured in a 5 d moving window from day 1 to day 13 and then were investigated when the 5 d linear slope increased rapidly by comparing with the values of days 1–4; it was on day 6 and day 9 for M1 and PFC, respectively (Extended Data Fig. 6e). We repeated this using different sizes of windows, that is, from 3 d to 7 d, and found that the linear slope significantly increased at day 6 for M1 and day 9 for PFC whichever window size was used. Thus, we focused on the trends of linear changes over time in the two distinct periods, for example, days 1–5 versus days 6–13 for M1 in each animal. For the days 1–5, we used all 15 tertiles to estimate linear slope; however, for the days 6–13, we used the tertiles from day 6 to the maximum-level tertile to focus on the rising phase and excluding the plateau phase at the latest period of motor learning. We repeated the same process for the PFC–M1 SO coupling (Figs. 1d,e) and the changes in success rate (Fig. 1f) but using all data points.

To test the effect of the rate of PFC SOs in the transitions of the PFC–M1 SO coupling, we recalculated the PFC–M1 SO coupling after subsampling the PFC SOs (Extended Data Fig. 2d). For the observed PFC SOs in a single session (for example, 425 events), 100 PFC SOs (this subsample number was fixed across sessions over time) were randomly subsampled, and then the PFC–M1 SO coupling was recalculated. In each session, this was repeated 1,000 times and the mean of those 1,000 measures was reported.

### Change-point analysis

Cross-areas coordination (PFC–M1 SO coupling and ΔSO–SWR coupling) was used to estimate two-stage transition time points during the long-term motor learning in Fig. 2f. We also performed ‘change-point analysis’ using the MATLAB function ‘findchangepts’. This finds a change-point at which some statistical property of a signal changes abruptly^68^ (single animal example in Extended Data Fig. 3e). The chosen statistic (type of change to detect) was the mean with setting to find a single change-point over 13 d data; the algorithm minimizes the total residual error from the best horizontal level of the mean for each stage. The stage transitions, that is, abrupt change based on PFC–M1 SO coupling and ΔSO–SWR coupling, were at 6.83 ± 0.17 d (mean ± s.e.m.) across the six animals.

### Quantification and statistical analyses

The figures show mean ± s.e.m.; if this was not the case, we specifically indicated so. Parametric statistics were generally used in this study (LME, *t*-tests, linear regression, Pearson’s correlation or otherwise stated), and were implemented within MATLAB. A ‘hierarchical nested statistics approach’ of LME (using the MATLAB function ‘fitlme’) was used for the comparison of SWRs rates, SOs rates and NREMS periods, in the main text and in Extended Data Fig. 1e; for the comparisons across days in Fig. 2b–d and Extended Data Figs. 2b, 3b,c, 4b, 5d, 6c, 7c,d and 8d; for the success rate, ΔSO–SWR coupling and neural trajectory fidelity in Fig. 6c; and for the changes in linear slope in Fig. 1e and Extended Data Figs. 2c and 6e. This was done to account for the repeated measures per animal; thus, this statistical approach ensured that the group level statistic accounted for sessions per animal and did not treat them as statistically independent samples. We fit random effects (for example, rats) specified as an intercept for each group and reported fixed effects representing population parameters to compare (for example, pre-hand-switch versus post-hand-switch). Adding random effects to a model recognizes correlations within sample subgroups (for example, rat) and extends the reliability of inferences beyond the variability across multiple rats. The fixed effects were tested for *P* values of the linear regression slope coefficients associated with two comparing conditions. The random effects and fixed effects parameters used are as follows: Fig. 2c,d and Extended Data Figs. 2b,c, 3c and 6e, random: rat, fixed: day; Figs. 2b,c and 3e and Extended Data Figs. 1e, 4b, 5d, 6c, 7c and 8d, random: rat, fixed: phase; Fig. 6c, random: rat, fixed: hand-switch. In these figures, the mean in each experimental session was used as the response parameter and two categories of the comparing conditions were used as the predictor parameter. For the comparison between two conditions in Extended Data Fig. 7a, we used two-sided two-sampled *t*-test. For the comparison between two paired conditions in Figs. 1e,f, 2b,f, 3e, 4a,d and 5d,e and Extended Data Figs. 3a and 8e–h, we used two-sided paired *t*-test.

We used traditional linear regression or correlation to evaluate the relationship from the motor learning period to the PFC–M1 SO coupling in Fig. 1d, the behavioural performance in Fig. 1f and the neural trajectory fidelity in Fig. 3d, as well as the relationship between behaviour and PFC–M1 SO coupling in Fig. 1g. For the comparison between distributions, we used Kolmogorov–Smirnov test in Fig. 5c. We also used logistic regression to illustrate the two-stage distinction of motor learning in Fig. 2g. Logistic regression requires predesignated parameter values for the predicting conditions, for example, tagging the stage I versus the stage II for each session. To tag the stage I versus stage II, we used the median of rise time (midpoint of sigmoid function fit) of PFC–M1 SO coupling and the drop time (midpoint of sigmoid function fit) of ΔSO–SWR coupling; this has been termed the ‘grand-midpoint’. We, thus, tagged the sessions before the grand-midpoint as stage 1 and the sessions after the grand-midpoint as stage II in each animal; the grand-midpoint occurred near days 7–8 across six animals. Then logistic regression was performed with the stage-tagged sessions to predict two distinct stages as a function of ΔSO–SWR coupling and PFC–M1 SO coupling.

### Reporting summary

Further information on research design is available in the Nature Portfolio Reporting Summary linked to this article.

## Online content

Any methods, additional references, Nature Portfolio reporting summaries, source data, extended data, supplementary information, acknowledgements, peer review information; details of author contributions and competing interests; and statements of data and code availability are available at 10.1038/s41586-022-05533-z.

## Supplementary information

Reporting Summary

## Data Availability

Data used in this manuscript are available at https://zenodo.org/deposit/7226711.

## Code availability

Custom MATLAB codes for the detection of SOs, spindles and SWRs are available at our GitHub repository (https://github.com/kimjack0/Sleep_Oscillation_Detection).
