# Supplementary material for: Cortical–hippocampal coupling during manifold exploration in motor cortex
Source: Nature. 2022 Dec 14;613(7942):103–10. doi: 10.1038/s41586-022-05533-z (PMC9812770; doi:10.1038/s41586-022-05533-z)
Supplement: Supplementary file 1 — Reporting Summary [file 41586_2022_5533_MOESM1_ESM.pdf]

nature portfolio

Corresponding author(s): Karunesh Ganguly

Last updated by author(s): Dec 6, 2022

Reporting Summary

Nature Portfolio wishes to improve the reproducibility of the work that we publish. This form provides structure for consistency and transparency in reporting. For further information on Nature Portfolio policies, see our Editorial Policies and the Editorial Policy Checklist.

Statistics

For all statistical analyses, confirm that the following items are present in the figure legend, table legend, main text, or Methods section.

n/a	Confirmed	
☐	☒	The exact sample size (*n*) for each experimental group/condition, given as a discrete number and unit of measurement
☐	☒	A statement on whether measurements were taken from distinct samples or whether the same sample was measured repeatedly
☐	☒	The statistical test(s) used AND whether they are one- or two-sided<br>*Only common tests should be described solely by name; describe more complex techniques in the Methods section.*
☐	☒	A description of all covariates tested
☐	☒	A description of any assumptions or corrections, such as tests of normality and adjustment for multiple comparisons
☐	☒	A full description of the statistical parameters including central tendency (e.g. means) or other basic estimates (e.g. regression coefficient) AND variation (e.g. standard deviation) or associated estimates of uncertainty (e.g. confidence intervals)
☐	☒	For null hypothesis testing, the test statistic (e.g. *F*, *t*, *r*) with confidence intervals, effect sizes, degrees of freedom and *P* value noted<br>*Give P values as exact values whenever suitable.*
☒	☐	For Bayesian analysis, information on the choice of priors and Markov chain Monte Carlo settings
☐	☒	For hierarchical and complex designs, identification of the appropriate level for tests and full reporting of outcomes
☐	☒	Estimates of effect sizes (e.g. Cohen's *d*, Pearson's *r*), indicating how they were calculated

Our web collection on statistics for biologists contains articles on many of the points above.

Software and code

Policy information about availability of computer code

Data collection	We used the software package Synapse (TDT) for collecting electrophysiology and behavioral time stamp data. We also used custom Matlab (2020a) code for controlling the automated reach-box and collecting the behavioral video data.
Data analysis	We used MountainSort for offline sorting, and custom Matlab (2020a) code for the analysis of all other data. The sleep oscillation detection codes are deposited in GitHub (https://github.com/kimjack0/Sleep_Oscillation_Detection).

For manuscripts utilizing custom algorithms or software that are central to the research but not yet described in published literature, software must be made available to editors and reviewers. We strongly encourage code deposition in a community repository (e.g. GitHub). See the Nature Portfolio guidelines for submitting code & software for further information.

Data

Policy information about availability of data

All manuscripts must include a data availability statement. This statement should provide the following information, where applicable:

- Accession codes, unique identifiers, or web links for publicly available datasets
- A description of any restrictions on data availability
- For clinical datasets or third party data, please ensure that the statement adheres to our policy

The datasets reported in this work is available from the Lead Contact upon reasonable request.

Field-specific reporting

Please select the one below that is the best fit for your research. If you are not sure, read the appropriate sections before making your selection.

☒ Life sciences ☐ Behavioural & social sciences ☐ Ecological, evolutionary & environmental sciences

For a reference copy of the document with all sections, see nature.com/documents/nr-reporting-summary-flat.pdf

Life sciences study design

All studies must disclose on these points even when the disclosure is negative.

Sample size	No statistical methods were used to predetermine sample sizes, but our sample sizes are similar to those reported in the previous publications. All collected sessions were used and shown in this work. For behavioral and electrophysiology analysis, the significance of effect was assessed for each animal.
Data exclusions	All collected sessions were used and shown in this work without exclusion. Single units (neurons) spike clustering quality were assessed by automated MountainSort curation process and then assessed manually. Only clearly identifiable units were included if firing rate did not drift over the recording session, and that had good waveforms and a high signal-to-noise ratio.
Replication	All relevant behavioral and neural effects were present in all animals trained. Effects reported in neural data were consistent across animals, and significant at the appropriate group level within the nested structure of the data.
Randomization	In our reach-to-grasp task, the pellet locations were not randomized across animals, but were adjusted and fixed in each animal. It was to guarantee the best performance in each animal with dominant hand use, and derive stabilization of behavior regarding the key hypothesis of this study. The neural events were compared to the group of randomized events to guarantee that no bias resulted in the findings of this study.
Blinding	We did not have separate behavioral group allocations. Given the fact that this study involved a difficult behavioral task, with large-scale electrophyiology recordings in a challenging brain region, to guarantee that we had sufficient numbers of animals and neurons, researchers needed to know the behavioral performance and recording outcome in real-time.

Reporting for specific materials, systems and methods

We require information from authors about some types of materials, experimental systems and methods used in many studies. Here, indicate whether each material, system or method listed is relevant to your study. If you are not sure if a list item applies to your research, read the appropriate section before selecting a response.

Materials & experimental systems

n/a	Involved in the study	
☒	☐	Antibodies
☒	☐	Eukaryotic cell lines
☒	☐	Palaeontology and archaeology
☐	☒	Animals and other organisms
☒	☐	Human research participants
☒	☐	Clinical data
☒	☐	Dual use research of concern

Methods

n/a	Involved in the study	
☒	☐	ChIP-seq
☒	☐	Flow cytometry
☒	☐	MRI-based neuroimaging

Animals and other organisms

Policy information about studies involving animals; ARRIVE guidelines recommended for reporting animal research

Laboratory animals	Adult Long-Evans male rats (300-400 g)
Wild animals	n/a
Field-collected samples	n/a
Ethics oversight	The experimental protocol was approved by the Institutional Animal Care and Use Committee at the San Francisco VA Medical Center.

Note that full information on the approval of the study protocol must also be provided in the manuscript.

March 2021
